# Exploring molecular characteristics and interactions of blood stasis syndrome in ischemic heart failure by integrated multi-omics

**DOI:** 10.3389/fmolb.2025.1627849

**Published:** 2025-10-13

**Authors:** Aolong Wang, Jingjing Wei, Lijie Qiao, Xingyuan Li, Ming Li, Xinfeng Zhu, Yilin Zhang, Qifei Zhao, Rui Yu, Bin Li, Xinlu Wang, Mingjun Zhu

**Affiliations:** ^1^ Department of Cardiovascular Disease, The First Affiliated Hospital of Henan University of Chinese Medicine, Zhengzhou, China; ^2^ Collaborative Innovation Center of Prevention and Treatment of Major Diseases by Chinese and Western Medicine, Zhengzhou, China; ^3^ The First Clinical Medicine College, Henan University of Chinese Medicine, Zhengzhou, China; ^4^ Henan Evidence-based Medicine Center of Chinese Medicine, The First Affiliated Hospital of Henan University of Chinese Medicine, Zhengzhou, China; ^5^ Prevention and Control Center for Chronic Disease, The First Affiliated Hospital of Henan University of Chinese Medicine, Zhengzhou, China

**Keywords:** ischemic heart failure, blood stasis syndrome, multi-omics, traditional Chinese medicine, molecular characteristics

## Abstract

**Background:**

Ischemic heart failure (IHF) is one of the leading causes of death worldwide. In traditional Chinese medicine, blood stasis syndrome (BSS) is regarded as a core pathological feature of IHF. This study aims to clarify the main biological characteristics and underlying mechanisms of BSS in IHF.

**Methods:**

Using an integrated multi-omics strategy combining transcriptomics, proteomics, and targeted metabolomics, we systematically analyzed the molecular characteristics of BSS in patients with IHF from multiple perspectives. By integrating multi-omics data and correlating them with clinical parameters, we identified a core molecular network associated with BSS. Key targets within this network were further validated using iPRM and RT-qPCR. ROC curve analysis and responses to pharmacological intervention were employed to confirm the diagnostic and therapeutic potential of these core molecular targets.

**Results:**

A total of 435 differentially expressed genes, 176 differentially abundant proteins, and 40 differentially altered metabolites related to IHF with BSS were identified. Multi-omics analysis highlighted the involvement of the complement and coagulation cascade and B cell receptor signaling pathway. Moreover, targeted metabolomics suggested that metabolic pathways associated with BSS involve glycine, arginine, tryptophan metabolism, and fatty acid biosynthesis. Integrated multi-omics correlation analysis identified CD79A, CD79B, CD19, CD22, CR2, F2, F8, F9, C3, FN1, 5-hydroxyindolacetic acid, and 4-acetamidobutanoate as core molecular features, with significant correlations observed between these molecules and clinical indicators. iPRM and RT-qPCR validation confirmed that the expression trends of these core molecules are consistent with the sequencing results. Furthermore, cross-validation with Yiqi Huoxue formula intervention identified F2, F8, F9, and FN1 as four diagnostic and therapeutic targets for BSS.

**Conclusion:**

This multi-omics analysis reveals that immune dysregulation, inflammation, and impaired coagulation function are central to BSS in IHF. Key pathways involved include the complement and coagulation cascade as well as B-cell receptor signaling, with F2, F8, F9, and FN1 identified as potential diagnostic and therapeutic targets. These findings provide new molecular insights into BSS in IHF.

## Graphical Abstract

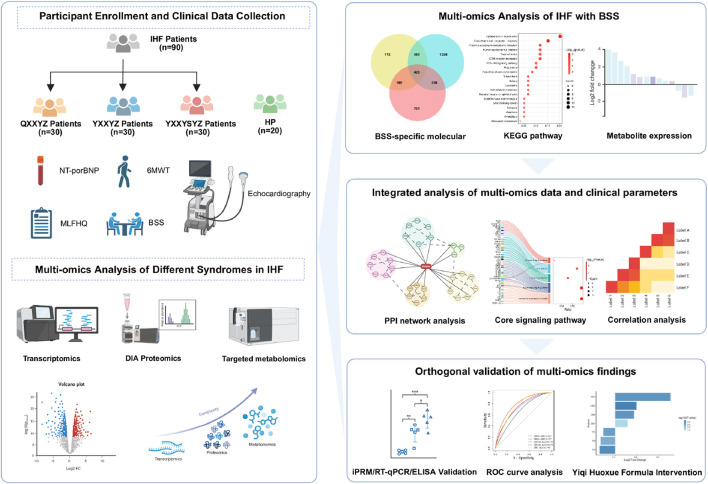

## 1 Introduction

Heart failure (HF) represents the end-stage manifestation of various cardiovascular diseases and remains a leading global cause of mortality. Worldwide epidemiological data indicate approximately 64 million HF cases, with China accounting for over 12 million affected individuals (Wang et al., 2021). Both prevalence and incidence rates exhibit age-dependent increases. Ischemic cardiomyopathy, characterized by myocardial fibrosis and consequent systolic/diastolic dysfunction due to chronic coronary atherosclerosis-induced ischemia (Pastena et al., 2024), represents the most prevalent etiology of HF (Vedin et al., 2017) and a major contributor to ischemic heart failure (IHF)-related mortality (Groenewegen et al., 2020). Emerging evidence highlights traditional Chinese medicine (TCM) as a promising therapeutic strategy for IHF management, demonstrating efficacy in decelerating disease progression and improving quality of life (Wan et al., 2023; Chen et al., 2023; Mao et al., 2020; Zhang et al., 2023). The syndrome differentiation approach, a cornerstone of TCM practice in China, classifies IHF patients into distinct subtypes (TCM syndromes) based on comprehensive clinical evaluations including symptoms, physical signs, and tongue manifestations (Bai et al., 2023; Su et al., 2014). While shared and divergent molecular mechanisms may underlie these syndrome classifications, However, current evidence remains insufficient to objectively elucidate the mechanistic basis underlying this differentiation.

Recent progress in multi-omics technologies, specifically transcriptomic, proteomic, and genomic methodologies, has markedly enhanced the mechanistic elucidation of diverse disease pathologies, as evidenced by recent studies (Aboumsallem et al., 2023; Liu X. et al., 2024; Zhang H. et al., 2022). Integrated with bioinformatics and network pharmacology, these approaches offer unprecedented opportunities to explore the molecular mechanisms underlying TCM syndrome differentiation. Accumulating studies have identified syndrome-specific biological signatures across diseases (You et al., 2021; Ding et al., 2023). For instance, multi-omics and network pharmacology analyses of two coronary heart disease syndromes revealed PON1 and ADIPOQ downregulation as biomarkers for the Cold Congealing and Qi Stagnation, while suppressed APOE and APOA1 expression characterized the Qi deficiency and blood stasis (Wu et al., 2021). Similarly, multi-omics profiling of type 2 diabetes patients stratified by stool consistency (dry vs. loose) demonstrated distinct gut microbiota composition, metabolic dysregulation, and insulin secretion patterns between subgroups (Guo et al., 2024), providing objective evidence for TCM syndrome classification. In TCM clinical practice, IHF patients are categorized into three therapeutic syndromes characterized by Qi deficiency with blood stasis syndrome (QXXYZ), Yang deficiency with blood stasis syndrome (YXXYZ), or Yang deficiency with blood stasis accompanied and fluid retention syndrome (YXXYSYZ). Among these, blood stasis syndrome (BSS) is considered a common characteristic of all IHF syndromes and is regarded as the core of IHF pathogenesis (Leung et al., 2023). However, the biological basis of BSS in IHF remains mechanistically undefined at the molecular level. The systematic integration of transcriptomic, proteomic, and targeted metabolomic profiling could delineate syndrome-specific molecular signatures, thereby advancing our understanding of this core pathological process in IHF.

In this study, we collected data from 90 IHF patients, including 30 patients each with QXXYZ, YXXYZ, and YXXYSYZ, along with 20 healthy participants (HP). Standardized clinical information collection and a serum sample repository were established. Using high-resolution RNA sequencing, quantitative proteomics, and targeted metabolomics (UHPLC-QQQ-MS/MS), we identified differentially expressed genes (DEGs), differentially expressed proteins (DEPs), and differentially metabolites (DMs) for the three syndromes compared to the HP. To elucidate the core pathobiological features of IHF with BSS, a comprehensive molecular regulatory network was constructed. In the validation phase, qPCR analysis confirmed transcriptional consistency of hub genes, complemented by PRM-based quantification of hub proteins within the protein-protein interaction (PPI) network. Additionally, we conducted a pharmacological intervention using the Yiqi Huoxue (YQHX) formula, analyzing multi-omics alterations before and after treatment to perform a reverse validation of the molecular mechanisms underlying BSS. This study systematically reveals the biological landscape of different IHF syndromes and the molecular network of BSS using a multi-omics cross-validation approach.

## 2 Materials and methods

### 2.1 Study design participants recruitment

The study protocol was registered in April 2022 with the Chinese Clinical Trial Registry (ChiCTR2200058314, accessible at: https://www.chictr.org.cn/). The research was approved by the Ethics Committee of the First Affiliated Hospital of Henan University of Chinese Medicine (approval number: 2021HL-178). Between June 2022 and July 2023, IHF patients were recruited from the Cardiac Center of the First Affiliated Hospital of Henan University of Chinese Medicine, and HP were recruited from the Health Checkup Center. All participants provided written informed consent.

### 2.2 Participants

Diagnostic criteria for IHF patients conformed to both the 2018 Chinese Society of Cardiology guidelines for heart failure management and the 2022 American Heart Association/American College of Cardiology/Heart Failure Society of America guidelines (Heidenreich et al., 2022). TCM syndrome differentiation strictly adhered to the Traditional Chinese Medicine Guideline for Diagnosis and Treatment of Chronic Heart Failure (2022) (Project Group of Traditional Chinese Medicine Guideline for Diagnosis and Treatment of Chronic Heart Failure, 2023). The TCM syndrome types were diagnosed by physicians based on a comprehensive evaluation of symptoms, physical signs, tongue appearance, and pulse condition, with specific diagnostic criteria provided in Supplementary Table S1. To ensure diagnostic consistency, three independent senior chief physicians independently evaluated the TCM syndrome classification of each case based on established guidelines. The final diagnosis was determined through a cross-validation process to achieve consensus.

#### 2.2.1 Included criteria

(1) Age between 40 and 80 years; (Pastena et al., 2024); Participants meet the diagnostic criteria for IHF and are diagnosed with TCM syndromes of QXXYZ, YXXYZ, or YXXYSYZ; (Vedin et al., 2017); New York Heart Association heart function class I–IV; (Groenewegen et al., 2020); Willingness to voluntarily sign a written informed consent form; (Wan et al., 2023); The BSS score ≥8.

#### 2.2.2 Excluded criteria

(1) Pulmonary embolism, acute coronary syndrome, or acute cerebrovascular diseases; (Pastena et al., 2024); Other cardiac conditions, such as valvular heart disease, severe valvular abnormalities, myocardial diseases, congenital heart diseases, or cor pulmonale; (Vedin et al., 2017); Liver and/or kidney dysfunction, malignant tumors, or autoimmune diseases; (Groenewegen et al., 2020); Participation in other studies within the past month; (Wan et al., 2023); Pregnant or lactating women.

### 2.3 Clinical data collection

Serum N-terminal pro-B-type natriuretic peptide (NT-proBNP) levels were quantitatively measured using the colloidal gold immunochromatography technique (Getein Biotech Kit, Nanjing, China). Left ventricular ejection fraction (LVEF) was assessed using the color Doppler ultrasound diagnostic device (GE Vivid E95, United States) with the two-dimensional Simpson method. The 6-min walk distance was determined using the standardized 6-min walk test (6MWT), performed by trained researchers. Multidimensional assessment was conducted using the Minnesota Living with Heart Failure Questionnaire (MLHFQ). The BSS scale (Supplementary Table S2) was used to evaluate the severity of BSS. Laboratory tests included lipid profile, fasting blood glucose, coagulation function, complete blood count, liver function, renal function, and routine urine analysis. All laboratory tests were performed using a standardized biochemical analysis system.

### 2.4 RNA-seq-based transcriptomic study

Fasting venous blood samples (2.5 mL) were collected in PAXgene™ tubes (PreAnalytiX, China) and processed per manufacturer’s protocols. Total RNA extraction employed the PAXgene Blood miRNA Kit (PreAnalytiX, China), with quality assessment conducted using an Agilent 5,400 Bioanalyzer (Agilent Technologies, United States). High-throughput sequencing was performed on the Illumina NovaSeq 6,000 platform (Illumina, United States). Raw read counts were normalized across samples using the R package DESeq2, DEGs were identified using the thresholds of |log_2_ (fold change)| > log_2_ (1.2) and an adjusted P-value <0.05 (Benjamini-Hochberg correction). Functional enrichment analysis of Gene Ontology (GO) terms and Kyoto Encyclopedia of Genes and Genomes (KEGG) pathways was performed using R package “Clusterprofiler” and “DOSE”.

### 2.5 Data-independent-acquisition-based proteomic study

Venous blood samples (2 mL) collected in EDTA-anticoagulated tubes from fasting subjects were centrifuged at 3,000×g for 15 min at 4 °C. The supernatant was transferred to pre-labeled Eppendorf tubes, aliquoted, and stored at −80 °C. Protein extraction and processing followed manufacturer protocols. High-abundance proteins were depleted using the ProteoMiner™ Low-Abundance Protein Enrichment Kit (Bio-Rad, United States). Protein concentration was quantified via Bradford assay (Biyuntian, China). The desalted peptide fragments were subjected to identification using liquid chromatography-tandem mass spectrometry (LC-MS/MS) (for detailed methodology, refer to Supplementary S1). DEPs were identified using the thresholds of |log_2_ (fold change)| > log_2_ (1.2) and P < 0.05. Functional enrichment analysis of GO terms and KEGG pathways was performed using R package “Clusterprofiler” and “DOSE”.

### 2.6 Targeted metabolomics study

Venous blood samples were collected from fasting participants in the early morning using 2 mL EDTA anti-coagulation tubes. After centrifugation at 3,000×g for 15 min at 4 °C, the supernatant was transferred to new Eppendorf tubes, labeled, aliquoted, and stored at −80 °C. Metabolite extraction was performed following the manufacturer’s instructions. Metabolite identification and analysis were conducted using an ultra-high-performance liquid chromatography tandem mass spectrometry (UHPLC-MS/MS) system (ExionLC™ AD UHPLC-QTRAP 6500+, AB SCIEX Corp., Boston, 196 MA, United States). A detailed description of the methods can be found in Additional file (Supplementary S2). Targeted metabolite standards (including 92 amino acids, 62 aromatic compounds, 43 organic acids, 38 bile acids, 19 fatty acids, 26 carbohydrates, 24 indoles, 23 nucleosides, nucleotides, and analogs, 14 phenylpropanoids, 9 pyridines, and 128 other compounds) were used for analysis. Metabolomic data were processed using the MetaX software, followed by principal component analysis (PCA) and partial least squares discriminant analysis. The variable importance in projection was calculated for each metabolite. Statistical significance between groups was assessed using t-tests, and FC of metabolites were determined. DMs were identified using the thresholds of |log_2_ (fold change)| > log_2_ (1.2) and VIP >1. Pathway analysis was performed using the MetaboAnalyst platform (https://www.metaboanalyst.ca/).

### 2.7 PPI network construction

PPI analysis was conducted using the STRING database (https://string-db.org/) with a minimum required interaction score of 0.4, and unconnected nodes were removed to construct the interaction network. The resultant networks were then imported into Cytoscape (v3.10.2) for visualization, further optimization, and calculation of topological parameters. Core genes/proteins analysis and identification of key molecular features were performed using the cytoHubba plugin in Cytoscape.

### 2.8 Joint analysis of transcriptomics, proteomics, and metabolomics

Functional module integration of transcriptomic and proteomic data was achieved via ClueGO/CluePedia plugins. KEGG pathway and GO enrichment analyses of DEGs/DEPs associated with BSS syndrome were performed using the ClueGO and CluePedia plugins in Cytoscape 3.10.2, with a significance threshold of P ≤ 0.05. These analyses facilitated functional module integration of transcriptomic and proteomic data, thereby clarifying the key biological processes regulated by BSS syndrome. In addition, correlation analysis identified metabolites significantly associated with the core molecules (P < 0.05).

### 2.9 Real-time quantitative polymerase chain reaction (RT-qPCR)

Gene expression validation was performed using BlazeTaq™ SYBR® Green qPCR Mix 2.0 (GeneCopoeia Green, United States) with primer sequences detailed in Additional file (Supplementary Table S3). β-actin served as the endogenous normalization control. Relative mRNA expression levels were calculated via the 2^−ΔΔCt^ method, with triplicate measurements conducted for each sample to ensure experimental reproducibility.

### 2.10 Intelligent parallel reaction monitoring (iPRM) quantitative proteomic analysis

Protein samples were processed following standardized extraction, quantification, and quality control protocols prior to tryptic digestion and desalting. Data-independent acquisition mode was employed for peptide spectral acquisition, generating raw mass spectrometry files (.raw). Spectronaut™ performed protein identification and label-free quantification under isocratic chromatographic conditions matching initial DIA parameters. An iPRM target list was integrated into the mass spectrometer method editor to establish Full MS-PRM acquisition parameters.

### 2.11 Enzyme-linked immunosorbent assay (ELISA) assay

The levels of TNF-α (E-EL254H0109), IL-1β (E-EL-H0149), IL-6 (E-EL-H6156), VCAM-1 (E-EL-H5587), and ICAM-1 (E-EL-H6114) were measured using ELISA kits (Wuhan Elabscience Biotechnology Co., Ltd.) according to the manufacturer’s instructions.

### 2.12 Therapeutic cross validation of BSS specific molecular features through YQHX granules

Building on our previous research (Zhu et al., 2022), we conducted an intervention study using the YQHX granules to further validate the molecular basis of BSS through integrative cross-omics analyses. YQHX granules is a TCM prescription known for its effects in tonifying Qi, activating blood circulation, and resolving stasis. The detailed composition of YQHX granules is provided in the Additional file (Supplementary S3). It is recommended by guidelines for the TCM management of HF [20], particularly in patients diagnosed with QXXYZ. On the basis of standard Western medical therapy, patients previously enrolled with IHF and diagnosed with QXXYZ were administered YQHX granules orally (21.6 g, twice daily) for a 12-week period. Multi-omics analysis (including transcriptomics and proteomics) was conducted using pre- and post-intervention samples from QXXYZ patients for comparative analysis and integrated comparison with previously identified BSS-associated molecular signatures, providing cross-validation of the biological basis of BSS from both diagnostic and interventional perspectives.

### 2.13 Statistical analyses

Categorical variables are presented as counts (n) and percentages (%). Continuous variables with a normal distribution are expressed as mean ± SD, while non-normally distributed data are presented as median (IQR). Parametric tests included independent two-tailed t-tests and one-way ANOVA, with homogeneity of variance assessed using Levene’s test. Non-parametric comparisons were conducted using the Mann-Whitney U or Kruskal-Wallis H tests. Count data were compared between groups using the chi-square test or Fisher’s exact test. For differential expression analysis in multi-omics analyses, the Benjamini-Hochberg false discovery rate (FDR) correction was applied to adjust for multiple hypothesis testing. For correlation analyses between omics data and clinical parameters, Spearman correlation coefficients were calculated. To control for multiple comparisons, Benjamini-Hochberg FDR correction was applied where necessary. All visualizations were generated using GraphPad Prism 9.0 (GraphPad, CA, United States). Bioinformatics plots (PCA, volcano, enrichment, Venn, Sankey) were created via https://www.bioinformatics.com.cn (accessed 10 December 2024).

## 3 Results

### 3.1 Study population and baseline characteristics

This study enrolled 90 IHF patients from the First Affiliated Hospital of Henan University of Chinese Medicine, including 30 cases each of QYXYZ, YXXYZ, and YXXYSYZ. Twenty healthy controls were recruited from the same hospital’s health examination center between June 2022 and July 2023. Comparative analysis of the HP, QYXYZ, YXXYZ, and YXXYSYZ groups revealed no significant intergroup differences in age, sex, Body Mass Index (BMI), diastolic blood pressure (DBP), total cholesterol (TC), high-density lipoprotein cholesterol (HDL-C), thrombin time (TT), or fibrinogen (FIB) (Table 1). Notably, the HP group exhibited significantly lower systolic blood pressure (SBP) and fasting blood glucose levels compared to the IHF subgroups (*P* < 0.05), likely reflecting physiological characteristics of healthy individuals. In contrast, IHF patients demonstrated reduced TC and low-density lipoprotein cholesterol (LDL-C) levels (*P* < 0.05), potentially attributable to ongoing lipid-lowering therapy. Prothrombin time (PT) and activated partial thromboplastin time (APTT) showed significant disparities between IHF patients and HP group. In addition, there were no statistically significant differences in comorbidities and medication use among the different TCM syndrome of IHF patients (Supplementary Table S4).

**TABLE 1:** Demographic and clinical characteristics of the subjects.

Variable	HP (n = 20)	QXXYZ (n = 30)	YXXYZ (n = 30)	YXXYSYZ (n = 30)	*p* value
Age (years)	64.95 (9.65)	66.47 (8.44)	66.40 (7.47)	68.0 (8.53)	0.568
Gender (Male)	13 (65.00%)	27 (90.00%)	23 (76.67%)	22 (73.33%)	0.163
BMI	23.78 (3.65)	24.72 (3.74)	23.56 (2.85)	23.33 (3.11)	0.395
SBP	118.55 (17.80)	134.00 (23.33)	129.77 (21.27)	122.13 (16.13)	0.027
DBP	74.7 (10.56)	76.73 (14.33)	72.50 (14.17)	68.10 (9.18)	0.056
TG	1.10 (0.35)	1.40 (0.54)	1.34 (0.65)	1.56 (0.99)	0.156
TC	4.42 (0.64)	3.71 (1.04)	3.67 (1.03)	3.67 (0.59)	0.01
HDL-C	1.29 (0.20)	1.24 (0.27)	1.17 (0.27)	1.15 (0.26)	0.245
LDL-C	2.95 (0.53)	2.10 (0.76)	2.11 (0.68)	2.11 (0.51)	<0.001
GLU	5.04 (0.48)	6.68 (2.66)	6.55 (2.18)	6.43 (2.27)	0.047
PT	10.74 (0.55)	12.46 (1.60)	11.76 (2.13)	12.59 (2.30)	0.003
APTT	31.69 (2.73)	28.52 (4.38)	28.68 (3.95)	29.30 (4.27)	0.033
TT	15.31 (0.89)	15.51 (1.96)	18.59 (19.30)	15.07 (1.74)	0.514
FIB	3.22 (0.55)	3.05 (0.81)	3.13 (0.91)	3.27 (1.09)	0.812

Data are n (%) or mean ± SD; *p* values were determined by one-way ANOVA and χ^2^ test. BMI, body mass index; SBP, systolic blood pressure; DBP, diastolic blood pressure; TG, triglyceride; TC, total cholesterol; HDL-C, high-density lipoprotein cholesterol; LDL-C, low-density lipoprotein cholesterol; GLU, fasting blood glucose; PT, prothrombin time; APTT, activated partial thromboplastin time; TT, thrombin time; FIB, fibrinogen.

### 3.2 Transcriptomic, proteomic, and targeted metabolomic analysis of QXXYZ, YXXYZ, and YXXYSYZ in IHF

**FIGURE 1:**
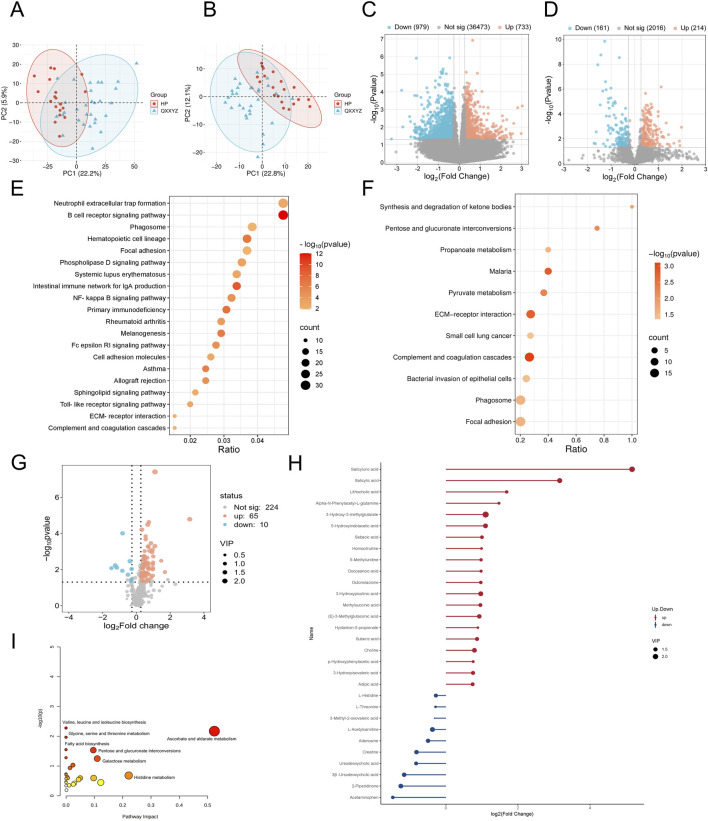
Transcriptomic, proteomic, and targeted metabolomic analysis of QXXYZ in IHF. **(A,B)** PCA plot of the transcriptomic and proteomics analysis of QXXYZ. **(C,D)** The volcano plots of DEGs and DEPs between QXXYZ and HP group. **(E,F)** KEGG pathways enriched with DEGs and DEPs of QXXYZ. **(G)** The volcano plots of DMs between QXXYZ and HP group. **(H)** DMs with Significant Changes of QXXYZ. **(I)** Functional enrichment analysis of identified DMs of QXXYZ.

**FIGURE 2:**
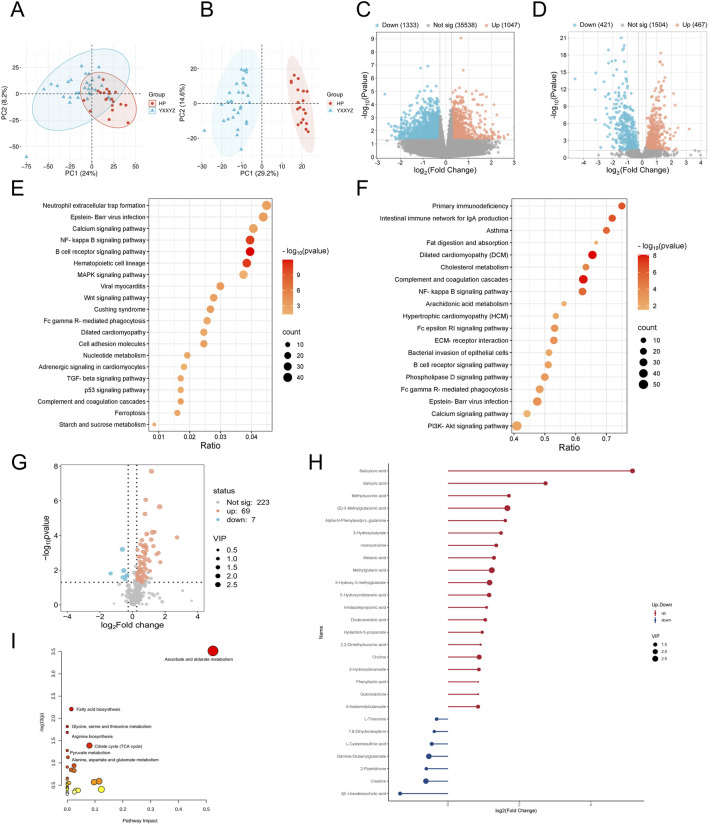
Transcriptomic, proteomic, and targeted metabolomic analysis of YXXYZ in IHF. **(A,B)** PCA plot of the transcriptomic and proteomics analysis of YXXYZ. **(C,D)** The volcano plots of DEGs and DEPs between YXXYZ and HP group. **(E,F)** KEGG pathways enriched with DEGs and DEPs of YXXYZ. **(G)** The volcano plots of DMs between YXXYZ and HP group. **(H)** DMs with Significant Changes of YXXYZ. **(I)** Functional enrichment analysis of identified DMs of YXXYZ.

**FIGURE 3:**
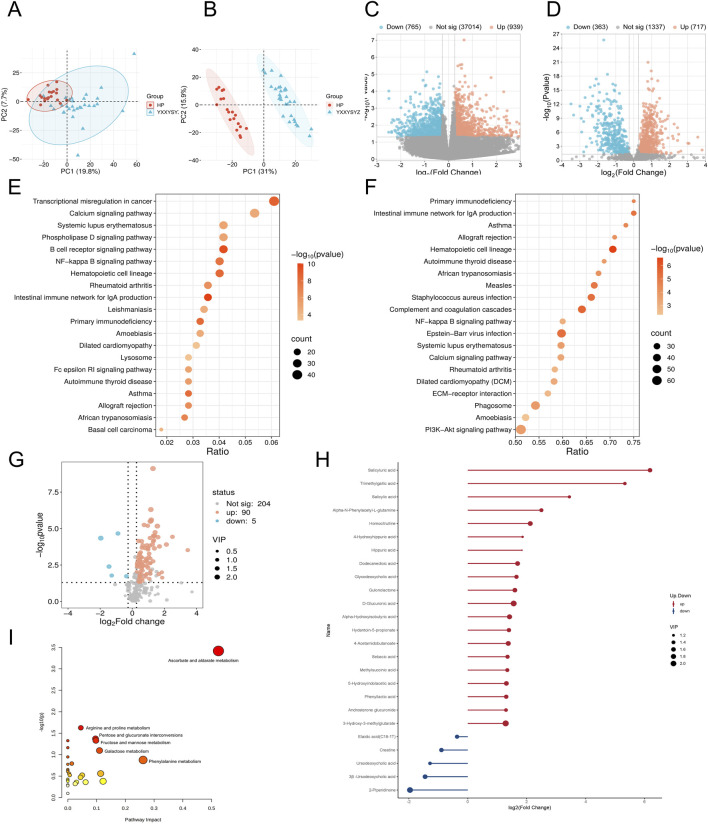
Transcriptomic, proteomic, and targeted metabolomic analysis of YXXYSYZ in IHF. **(A,B)** PCA plot of the transcriptomic and proteomics analysis of YXXYSYZ. **(C,D)** The volcano plots of DEGs and DEPs between YXXYSYZ and HP group. **(E,F)** KEGG pathways enriched with DEGs and DEPs of YXXYSYZ. **(G)** The volcano plots of DMs between YXXYSYZ and HP group. **(H)** DMs with Significant Changes of YXXYSYZ. **(I)** Functional enrichment analysis of identified DMs of YXXYSYZ.

Integrated multi-omics analysis demonstrated that QXXYZ, YXXYZ, and YXXYSYZ exhibit both common and distinct molecular characteristics. PCA revealed clear separations from the HP group in both transcriptomic and proteomic profiles across all three syndromes, indicating distinct molecular architectures (Figures 1A,B, 2A,B, 3A,B). The quantities of DEGs, DEPs, and DMs identified in each group are summarized in Table 2 and illustrated in Figures 1C,D,G, 2C,D,G, 3C,D,G. KEGG pathway enrichment analysis highlighted several overlapping pathways among the three subtypes, including the B cell receptor (BCR) signaling pathway, NF-κB signaling pathway, and complement and coagulation cascades (Figures 1E,F, 2E,F, 3E,F). These recurrent pathways reflect common mechanisms underlying immune dysregulation, inflammation, and coagulation abnormalities. Targeted metabolomics further confirmed significant metabolic alterations across all groups, with fatty acids, amino acids, and organic acids representing the major classes of disturbed metabolites (Supplementary Tables S5, S6, S7), Significantly upregulated and downregulated metabolites are presented in Figures 1H, 2H, 3H. Ascorbate and aldarate metabolism was identified as a consistently dysregulated metabolic pathway among all syndromes (Figures 1I, 2I, 3I). Despite these shared features, important differences were observed. QXXYZ exhibited significant enrichment in pathways such as pentose and glucuronate interconversions and ketone body metabolism. Concurrently, key metabolic disturbances were observed in the biosynthesis of valine, leucine, and isoleucine. These findings indicate that its characteristics may be associated with energy metabolic reprogramming (Matsuura et al., 2023; Cluntun et al., 2021; Foster et al., 2024). In contrast, YXXYZ was more strongly associated with pathways linked to myocardial injury and fibrosis, including dilated cardiomyopathy, TGF-β signaling, calcium signaling pathway, and PI3K-Akt signaling pathway, suggesting a more severe involvement of cardiac impairment and fibrotic remodeling (Luczak et al., 2020; Ruiz-Villalba et al., 2020; Ouwerkerk et al., 2023). This group also exhibited specific alterations in glycine, serine, and threonine metabolism. YXXYSYZ showed biological features highly reminiscent of YXXYZ, but with a distinct metabolic emphasis on pentose and glucuronate interconversions.

**TABLE 2:** The quantities of DEGs, DEPs, and DMs identified in each group.

Group	DEGs (UP/Down)	DEPs (UP/Down)	DMs (UP/Down)
QXXYZ	1,712 (733/979)	375 (214/161)	75 (65/10)
YXXYZ	2,380 (1047/1333)	888 (467/421)	75 (69/7)
YXXYSYZ	1,704 (939/765)	1080 (717/363)	95 (90/5)

DEGs, differentially expressed genes; DEPs, differentially expressed proteins; DMs, differential metabolites; QXXYZ, Qi deficiency with blood stasis syndrome; YXXYSYZ, Yang deficiency with blood stasis accompanied by fluid retention syndrome; YXXYZ, Yang deficiency with blood stasis syndromeSupplementary Information.

### 3.3 Transcriptomic, proteomic, and targeted metabolomic analysis of BSS in IHF

**FIGURE 4:**
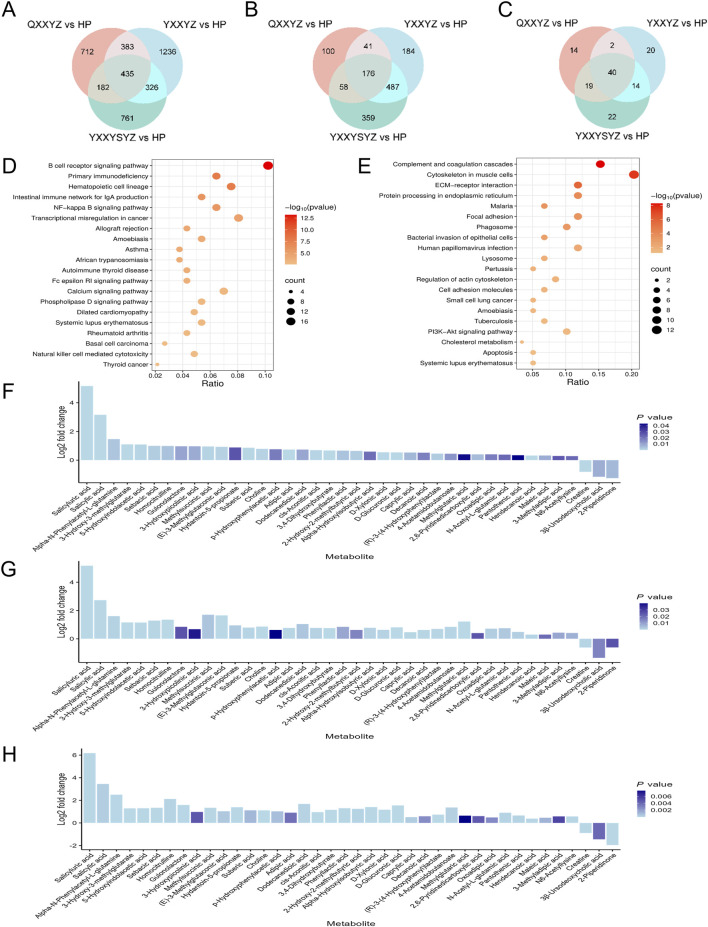
Transcriptomic, proteomic, and targeted metabolomic analysis of BSS in IHF. **(A–C)** Venn diagram identifying DEGs, DEPs, DMs associated with BSS. **(D)** KEGG pathways enriched with DEGs of BSS. **(E)** KEGG pathways enriched with DEPs of BSS. **(F–H)** Fold-changes and *P* values of DMs of QXXYZ, YXXYZ, YXXYSYZ.

To further elucidate the biological basis of BSS, we identified DEGs, DEPs and DMs related to IHF with BSS using Venn diagram analysis. Transcriptomic analysis identified 435 DEGs (Figure 4A). KEGG enrichment revealed that DEGs were significantly associated with immune and inflammatory pathways, particularly the BCR signaling pathway (Figure 4D). PPI network analysis further identified CD19, CD79A, CD79B, and CR2 as hub genes potentially involved in the pathogenesis of BSS (Supplementary Figure S1). Proteomic analysis identified 176 DEPs (Figure 4B), which were primarily enriched in pathways including the complement and coagulation cascades, and the PI3K/Akt signaling pathway (Figure 4E). PPI analysis highlighted FN1, C3, F2, F8, and F9 as central proteins, suggesting their pivotal roles in BSS-related pathological processes (Supplementary Figure S2). A total of 40 DMs were identified through metabolomic profiling (Figure 4C), with the majority classified as fatty acids (32.5%), and amino acids (15%). Among them, Alpha-N-Phenylacetyl-L-glutamine, 3-Hydroxy-3-methylglutarate, and 5-Hydroxyindolacetic acid showed a consistent upregulation trend, whereas Caprylic acid, 2-Piperidinone, and 3β-Ursodeoxycholic acid were commonly downregulated (Figures 4F–H). Pathway enrichment analysis using MetaboAnalyst revealed significant involvement of glycine, serine, and threonine metabolism (Choline, Creatine), arginine and proline metabolism (Creatine, 4-Acetamidobutanoate), and tryptophan metabolism (5-Hydroxyindoleacetate, 2-Oxoadipate) (Supplementary Figure S3).

### 3.4 Comprehensive analysis of the molecular interaction network of BSS in IHF and its correlation with clinical data

**FIGURE 5:**
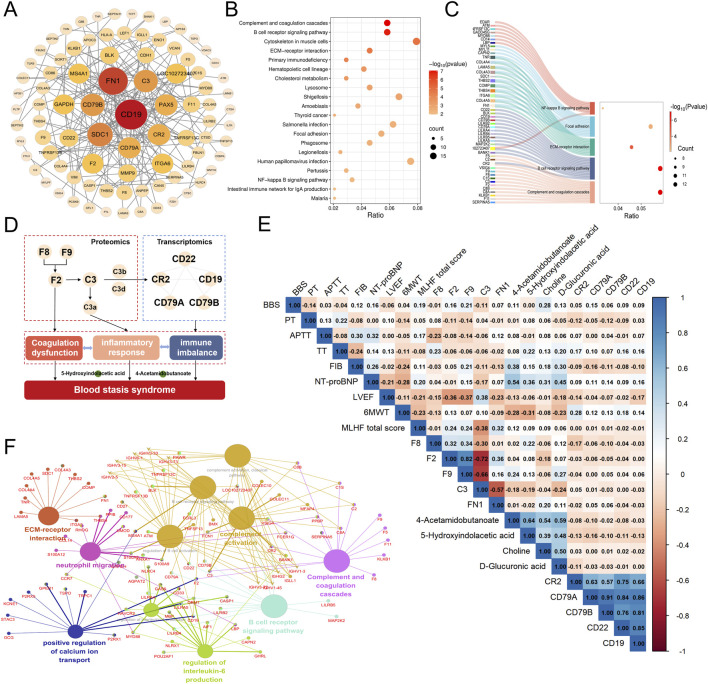
Comprehensive analysis of the molecular interaction network of BSS in IHF and its correlation with clinical data. **(A)** PPI Network of DEGs and DEPs of BSS. **(B)** KEGG pathways enriched with DEGs and DEPs of BSS. **(C)** Sankey Diagram of Enriched Pathways for Key Molecules of BSS. **(D)** Core Molecules and Functional Networks Underlying BSS Revealed by Multi-Omics Integration. **(E)** Heatmap of the Correlation Between Key Molecules and Clinical Data. **(F)** ClueGO analysis of GO and KEGG enrichment.

We conducted an integrated analysis of transcriptomic, proteomic, targeted metabolomic, and clinical indicator data to systematically elucidate the molecular regulatory network of BSS. Differential genes and proteins related to BSS were first uploaded to the STRING database, and the core molecular network was constructed using Cytoscape software. Key regulatory molecules, including CD19, CD22, CD79A, CD79B, C3, CR2, F2, F8, and F9, were identified (Figure 5A). KEGG pathway enrichment analysis and ClueGO network visualization revealed biological mechanisms associated with BSS, such as complement and coagulation cascades, BCR signaling pathway, and ECM-receptor interaction pathway (Figures 5B,F). A Sankey diagram further uncovered the relationships between core regulatory molecules and key pathways (Figure 5C). In addition, through correlation analysis, we identified 17 metabolites significantly associated with core molecules (P < 0.05) (Supplementary Table S8), including 5-Hydroxyindolacetic acid, D-Glucuronic acid, 4-Acetamidobutanoate, and Choline. The pathogenesis of BSS is primarily driven by sustained activation of the complement and coagulation cascades, which disrupts BCR signaling and subsequently triggers immune imbalance, hyperinflammation, coagulation dysfunction, and metabolic disturbances (Figure 5D). These integrated alterations constitute the core pathological features of BSS.

As core molecules related to BSS, CR2, F2, F8, F9, FN1, 5-Hydroxyindolacetic acid, and 4-Acetamidobutanoate were further analyzed for their correlations with clinical indicators. The results showed that CR2 expression was positively correlated with the 6MWT. F8 exhibited a positive correlation with APTT, And F9 was positively correlated with BSS score, while F2 and F9 were significantly negatively correlated with LVEF and positively correlated with MLHFQ scores. Additionally, 5-hydroxyindolacetic acid, D-glucuronic acid, 4-acetamidobutanoate, and choline were positively correlated with NT-proBNP levels. Notably, 5-hydroxyindolacetic acid and 4-acetamidobutanoate were significantly negatively correlated with 6MWT (Figure 5E). These findings highlight the potential involvement of these core molecules in the pathophysiology of BSS and their association with key clinical parameters.

**FIGURE 6:**
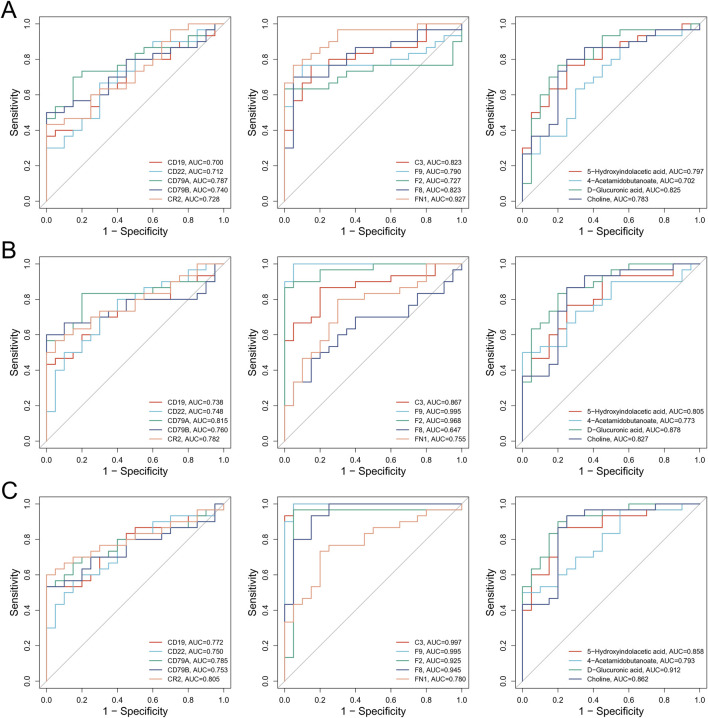
Comprehensive analysis of the molecular interaction network of BSS in IHF and its correlation with clinical data. **(A)** ROC Curve Analysis of Core Molecules Associated with BSS in QXXYZ. **(B)** ROC Curve Analysis of Core Molecules Associated with BSS in YXXYZ. **(C)** ROC Curve Analysis of Core Molecules Associated with BSS in YXXYSYZ.

To evaluate the diagnostic potential of key molecules identified through multi-omics analyses for the diagnosis of BSS, receiver operating characteristic (ROC) curve analysis was conducted for candidate biomarkers. The results revealed that the diagnostic performance of key proteins C3, F2, F8, F9, and FN1 was superior to that of BSS associated genes and metabolites (Figure 6), suggesting a higher clinical value of proteomic markers in identifying BSS. Specifically, in the QXXYZ group, FN1 (AUC = 0.927), C3 (AUC = 0.823), and F8 (AUC = 0.823) exhibited the highest discriminative power as individual biomarkers (Figure 6A). In the YXXYZ and YXXYSYZ groups, F9 (AUC = 0.995) and F2 (AUC = 0.968) showed high diagnostic accuracy. C3 (AUC = 0.997) in the YXXYSYZ group also exhibited superior diagnostic efficacy (Figures 6B,C). To further improve the diagnostic accuracy of the model, the integration of five key proteins C3, F2, F8, F9, and FN1 resulted in a combined prediction that achieved an AUC of 0.971 for QXXYZ. This combined model demonstrated a marked improvement over individual biomarkers, indicating that the multi-marker approach effectively enhances the diagnostic sensitivity and specificity for BSS.

### 3.5 Validation of key molecules

**FIGURE 7:**
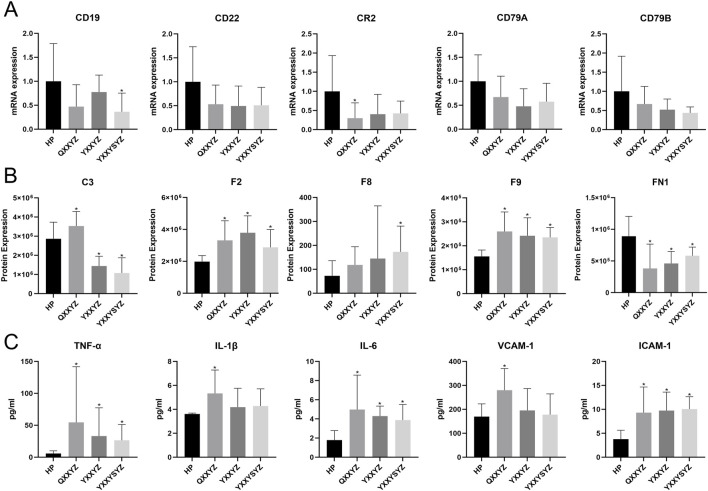
Validation of Core Molecules Associated with Blood Stasis Syndrome. **(A)** The mRNA expression validated by RT-qPCR. **(B)** The expression of Core proteins verified by iPRM. **(C)** The level of TNF-α, IL-1β, IL-6, VCAM-1, ICAM-1 in each group was detected by ELISA kit. n = 10 in each group. *p < 0.05 vs the HP group.

By additionally collecting 10 healthy participants and 10 patients for each of the three different syndrome types, key molecules were validated using RT-qPCR and iPRM techniques. RT-qPCR results showed that, compared to healthy controls, the expression levels of CR2 in the QXXYZ group and CD19 in the YXXYSYZ group were significantly reduced (p < 0.05). The expression levels of CD22, CD79A, and CD79B in all three syndrome types showed a downward trend, but the differences were not statistically significant (Figure 7A). PRM analysis revealed that the concentrations of F2 and F9 in the BSS group were significantly higher than in the healthy control group (p < 0.05). The F8 concentration in the YXXYZ group was significantly higher than that in the control group (p < 0.05), while QXXYZ and YXXYZ showed an upward trend, although the differences were not statistically significant. Furthermore, the expression level of C3 was elevated in the QXXYZ group, while it decreased in the YXXYZ and YXXYSYZ groups (Figure 7B), consistent with the expression trends observed in sequencing data for the relevant genes and proteins. ROC curve analysis revealed that F2 (AUC = 0.917), F9 (AUC = 0.913), and FN1 (AUC = 0.880) exhibited the highest diagnostic performance, with all other AUC values greater than 0.65 (Supplementary Figures S4, S5). Additionally, we further validated the levels of TNF-α, IL-1β, IL-6, ICAM-1, and VCAM-1. Compared to the control group, the expression levels of TNF-α, IL-6, and ICAM-1 were significantly elevated (p < 0.05). Meanwhile, IL-1β and VCAM-1 also showed an increasing trend, but only the QXXYZ group exhibited statistically significant differences (Figure 7C).

**FIGURE 8:**
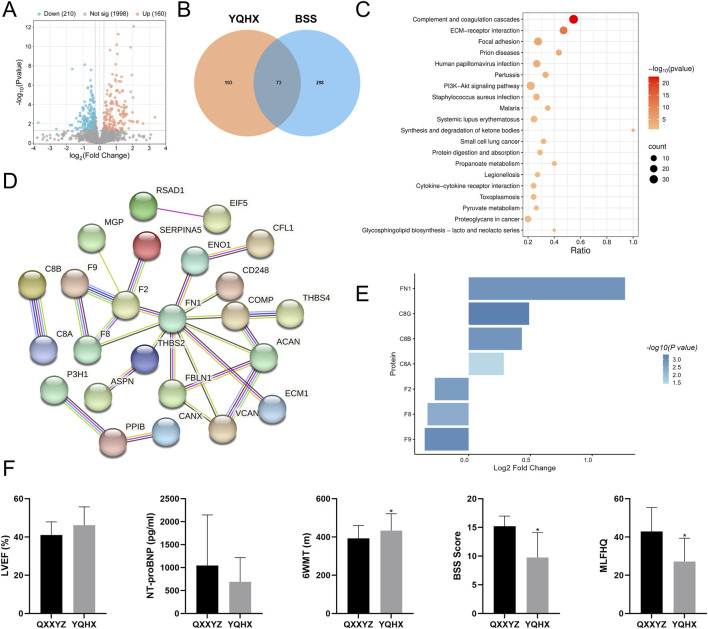
Multi-Omic Cross Validation of YQHX Granules treatment in IHF with QXXYZ. **(A)** DEPs identified after YQHX granules treatment in IHF with QXXYZ. **(B)** Integrative Analysis Identifies Overlapping Proteins between DEPs identified after treatment and BSS Associated DEPs. **(C)** KEGG Pathway Enrichment Analysis of DEPs after treatment. **(D)** PPI network of overlapping DEPs. **(E)** Expression changes of key proteins after treatment. **(F)** Clinical efficacy indicators after treatment. ^*^ *p* < 0.05 vs the QXXYZ group.

To validate our findings, we analyzed transcriptomic and proteomic data from 25 previously enrolled patients diagnosed with QXXYZ who received treatment with the YQHX granules. Transcriptomic profiling revealed 289 DEGs, predominantly enriched in NETs formation and BCR signaling pathways (Supplementary Figure S7), suggesting a potential immunomodulatory effect of YQHX granules. Proteomic analysis further identified 370 DEPs (Figure 8A), which were enriched in pathways associated with the pathophysiological features of BSS, including complement and coagulation cascades and ECM-receptor interactions (Figure 8C). Integrative analysis intersecting post-intervention DEPs with established BSS-associated proteins identified 73 overlapping candidates (Figure 8B). Network analysis revealed a tightly interconnected module centered on coagulation factors F2, F8, F9, and fibronectin FN1 (Figure 8D), suggesting their dual role as pathological hubs and therapeutic targets.

Following YQHX treatment, a significant downregulation in the expression of F2, F8, and F9 was observed, while FN1 expression was upregulated (Figure 8E), further supporting their role as key molecular components in the pathogenesis and therapeutic response of BSS. Notably, B cell signaling components (C3, CR2, CD19, CD79A/B) exhibited stable expression post-intervention (Supplementary Table S9). Clinical parameter analysis provided additional evidence supporting the therapeutic efficacy of the intervention. After treatment, LVEF showed a trend toward improvement, while NT-proBNP levels decreased and 6MWT significantly increased. Furthermore, both BSS scores and MLHFQ scores were significantly reduced (Figure 8F).

## 4 Discussion

In the long-standing clinical practice of TCM, syndrome differentiation has always been considered the core of clinical diagnosis and treatment. It essentially represents a staged response pattern of the body’s interaction with internal and external pathogenic factors throughout the disease progression, while integrating the patient’s inherent traits and time-specific physiological and pathological features (Huang et al., 2024). The same principle applies to IHF, where, similar to modern medicine’s classification of acute HF into dry cold, wet cold, dry warm, and wet warm subtypes, TCM commonly classifies IHF into the syndromes QXXYZ, YXXYZ, and YXXYSYZ. BSS is a shared characteristic among these syndromes and is considered a persistent feature throughout the development of HF. Furthermore, BSS is also a critical syndrome in various other diseases, and in recent years, substantial research has focused on its biological basis (Li L. et al., 2020; Lan et al., 2024; Liu Y. et al., 2024). However, there is still a lack of research addressing the biological basis of blood stasis in IHF. Our study, for the first time, provides a comprehensive multi-omics perspective on the different syndrome types of IHF and, through integrated multi-omics analysis, further elucidates the specific molecular interaction network associated with BSS in IHF.

Our integrated multi-omics analysis reveals that the pathogenesis of BSS involves a coordinated dysregulation across multiple biological layers, likely driven by sustained activation of the complement and coagulation cascades. This process disrupts the homeostatic regulation of BCR signaling, leading to impaired B cell functionality. IHF is characterized by systemic inflammation that promotes innate and adaptive immune cell infiltration into the myocardium, a process underpinned by immunometabolic dysregulation (Andreadou et al., 2025). The complement system serves as a critical bridge between innate and adaptive immunity and collaborates with B cells in mediating inflammatory responses in HF (Wang et al., 2025). Following myocardial injury, autoantigen release activates B cells to produce autoantibodies, which together with complement components perpetuate myocardial inflammation, cell death, and fibrosis, thereby driving adverse cardiac remodeling (Fragasso et al., 2025). In IHF hearts, significant upregulation of coagulation factors, complement components, and innate immune pathways is observed, accompanied by a distinct “coagulation-inflammation” signature (Garlapati et al., 2023). Concurrent downregulation of CR2, CD19, CD22, CD79A, and CD79B impairs complement-B cell coupling, shifting complement activation toward pro-inflammatory pathways rather than adaptive immune regulation. This imbalance exacerbates chronic inflammation, endothelial injury, and fibrosis. Cox regression models adjusted for covariates in a cohort of 185 heart failure patients identified complement and coagulation-related proteins (C3, C7, C8, C9, F9) as significant predictors of disease progression (Duan et al., 2019). Consistent with this, proteomic profiles in HF with preserved ejection fraction and kidney injury are associated with complement activation, inflammation, fibrosis, and impaired cholesterol homeostasis (Salman et al., 2024). Previous metabolomic studies in ischemic cardiomyopathy reported elevated levels of serotonin and valine (Li M. et al., 2020), which may further contribute to immune and coagulation dysregulation. Notably, recent intervention studies demonstrate that inhibiting tissue factor with nematode anticoagulant protein c2 (NAPc2) effectively blunts the “coagulation-inflammation-fibrosis” axis post-myocardial infarction, thereby improving cardiac function and attenuating remodeling in IHF (Garlapati et al., 2023). Together, these findings underscore that immune, inflammatory, and coagulation abnormalities represent key pathological mechanisms in IHF, highlighting potential therapeutic targets to modulate this maladaptive cascade.

BSS is characterized by impaired blood flow and internal blood stasis, leading to local circulatory dysfunction and clinical signs such as dark purplish lips, cyanotic tongue, and sublingual stasis (Qiu and Xu, 2022). The coagulation cascade not only alters hemorheological properties but also regulates endothelial dysfunction, inflammation, and fibrosis via protease-activated receptors (Friebel et al., 2019), thereby exacerbating microvascular disturbances in line with the pathophysiology of blood stasis in TCM. Additionally, the complement system and BCR signaling jointly contribute to immune-inflammatory responses in IHF, promoting endothelial injury and coagulopathy, which aligns with the TCM concept of Blood stasis obstructing circulation (Erdei et al., 2021; Lu et al., 2021). YQHX granule-targeted network analysis pinpointed F2, F8, F9, and FN1 as dual pathological effectors and therapeutic nodes. Although transcriptomic data post-YQHX intervention showed enrichment in BCR signaling, no significant changes were observed in the expression of CR2, CD19, CD79A, or CD79B, suggesting these immune markers may represent stable molecular characteristics of BSS rather than short-term therapeutic targets. Notably, previous studies of BSS in coronary artery disease and stroke also identified enrichment of these pathways (Wu et al., 2021; Liu Y. et al., 2024), supporting the hypothesis that BSS may share a common biological basis across multiple disease contexts.

C3, F2, F8, and F9 were identified as core proteins within the complement and coagulation cascades, with PPI network analysis suggesting their potential regulatory roles in the pathogenesis of BSS in IHF. Previous studies have confirmed the involvement of complement and coagulation proteins in HF progression through risk models adjusted for clinical covariates (Egerstedt et al., 2019). In our study, correlation analyses revealed that F2 and F9 levels were positively associated with BSS scores and negatively correlated with LVEF, indicating their potential value in reflecting both syndrome severity and cardiac function. Additionally, F2, F8, and F9 showed favorable diagnostic performance, suggesting their utility as candidate biomarkers for BSS. C3, another key molecule, has been shown to deposit in infarcted myocardium and contribute to ongoing injury in non-infarcted regions (Sintou et al., 2020). C3 knockout alleviated right ventricular dysfunction and fibrosis in animal models (Ito et al., 2022), likely by promoting fibroblast activation and ECM remodeling via the C3a-C3aR axis. However, clinical studies have reported C3 levels decline by approximately 53% in acute and 30% in chronic HF (Morales et al., 2020), reflecting its potential dynamic variation across disease stages. In our study, significant differences in C3 levels were observed among various TCM syndromes, suggesting that syndrome differentiation may be associated with the severity of IHF. Furthermore, C3 levels showed a positive correlation with LVEF and a negative correlation with MLHFQ scores, demonstrating good diagnostic performance and potential clinical value in identifying BSS. Given the localized activation of complement components within target organs, peripheral C3 levels may not fully reflect tissue-level activity (Frey et al., 2013). Considering the pathological products of complement activation may enhance the clinical interpretation of complement involvement in IHF.

The BCR complex components CD19, CD22, CD79A, and CD79B are critical mediators of antigen recognition and signal transduction, maintaining B cell immune homeostasis through coordinated molecular interactions (Erdei et al., 2021). B cell dysregulation and impaired regulatory B cell function have been implicated in HF pathophysiology (Lu et al., 2021). Although our data suggest enrichment of the BCR signaling pathway in BSS, its correlation with clinical indicators and diagnostic performance was limited, likely stemming from multifactorial pathway regulation. Notably, CR2 co-signaling with BCR can suppress IL-6 production (Kovács et al., 2021). In this study, we observed downregulation of CD19, CD22, CD79A, CD79B, and CR2 alongside elevated IL-6 levels, indicating that restoring B cell homeostasis and attenuating inflammation may represent therapeutic targets in IHF. Overall, these findings suggest that sustained activation of the complement and coagulation cascades leads to inflammatory and thrombotic disturbances, which may in turn impair B cell function and contribute to immune dysregulation. The interplay between immune inflammation and Coagulation dysfunction may represent a core pathological mechanism underlying BSS in IHF.

Targeted metabolomic analysis identified 40 metabolites associated with BSS, primarily comprising fatty acids (32.5%) and amino acids (15%). Consistent with previous studies in LAD-ligation HF rat models showing elevated amino acid and fatty acid levels in BSS (Qiu et al., 2014), our findings highlight disturbed metabolic pathways linked to inflammation and platelet activation. 5-Hydroxyindolacetic acid (5-HIAA), a terminal product of serotonin (5-HT) metabolism, was significantly elevated in BSS. Its accumulation indicates abnormal activation of the tryptophan–5-HT pathway. During platelet activation, 5-HT is converted to 5-HIAA by monoamine oxidase, which can stimulate neutrophils and amplify inflammation (Rieder et al., 2021; Shajib et al., 2015). Moreover, increased tryptophan catabolism has been linked to poor outcomes in chronic HF with low grade inflammation (Wang et al., 2024). It is also worth noting that the tryptophan metabolic pathway and 5-HIAA levels may be associated with sustained inflammation and the production of IL-10 (Lanis et al., 2017; Rosser et al., 2020). In our study, 5-HIAA levels were significantly correlated with clinical biomarkers, suggesting its potential role in mediating the crosstalk between coagulation and inflammatory responses. On the other hand, elevated levels of 4-acetamidobutanoate reflect abnormalities in arginine metabolism. During immune-inflammatory activation, increased arginase activity redirects arginine metabolism toward ornithine production, suppressing nitric oxide synthesis and thereby promoting vasoconstriction, impaired blood flow, a hypercoagulable state, and local inflammation, which collectively contribute to the development of BSS. In our study, 4-acetamidobutanoate levels were positively correlated with F9 and NT-proBNP, and negatively correlated with 6MWT performance. In addition, a cohort study reported that elevated 4-acetamidobutanoate levels were associated with increased risk of HF hospitalization (Tahir et al., 2021). Notably, creatine, synthesized from glycine and arginine, was reduced in BSS compared to healthy controls. As a key metabolite involved in myocardial energy metabolism and contractility, reduced creatine may increase susceptibility to ischemic injury (McAndrew et al., 2023; Balestrino, 2021). These findings highlight a dysregulated metabolic network involving tryptophan, arginine, and glycine as a potential mechanistic basis of blood stasis syndrome. Among these metabolites, 5-HIAA and 4-acetamidobutanoate may serve as potential biomarkers bridging coagulation and inflammation, contributing to disease progression.

QXXYZ, YXXYZ, and YXXYSYZ represent three distinct subtypes of BSS in IHF, each characterized by unique underlying biological mechanisms, reflecting the dynamic interplay of Qi deficiency, Yang deficiency, and blood stasis in the progression of IHF (Project Group of Traditional Chinese Medicine Guideline for Diagnosis and Treatment of Chronic Heart Failure, 2023). Multi-omics analysis revealed that QXXYZ is associated with NET formation, NF-κB signaling, pyruvate metabolism, and ketone body synthesis and degradation, indicating dysregulation of energy metabolism and immune-inflammatory responses. Qi deficiency is thought to impair the driving force of circulation, predisposing to blood stasis. The observed disruption in pyruvate metabolism suggests impaired mitochondrial oxidative function and ATP depletion (Lopaschuk et al., 2021), providing a biological basis for the concept of Qi insufficiency. Metabolic reprogramming toward glycolysis under energy stress may promote NET formation (Fernandez-Caggiano and Eaton, 2021; Stojkov et al., 2022). NETs, in turn, serve as platforms for complement activation and scaffolds for thrombus formation, contributing to microcirculatory dysfunction and a prothrombotic state (de Bont et al., 2019). In contrast, both YXXYZ and YXXYSYZ share similar transcriptomic and proteomic profiles, with enrichment in NF-κB signaling, calcium signaling, and ECM–receptor interaction pathways. Clinically, these two subtypes often present with overlapping symptoms, likely due to their common origin from longstanding Qi deficiency progressing to Yang deficiency. Persistent NF-κB activation promotes chronic low-grade inflammation, characterized by overexpression of TNF-α, IL-1β, and IL-6, which contributes to cardiomyocyte apoptosis and myocardial fibrosis (Du et al., 2025; Zhang Q. et al., 2022). Calcium signaling dysregulation further exacerbates cardiomyocyte injury (Mo et al., 2021). Collectively, these alterations lead to immune imbalance, aggravating blood stasis and accelerating HF progression.

In conclusion, our findings provide new insights into the biological basis of BSS in IHF and the evolution of TCM syndromes. However, several limitations must be acknowledged. First, we conducted only clinical multi-omics analyses and validated the findings solely in QXXYZ patients following YQHX intervention, without further validation across other TCM syndrome subtypes. Second, due to the targeted nature of the metabolomics approach, integration with transcriptomics and proteomics was limited to correlation analyses. Third, the relatively small sample size, restricted geographic distribution of participants, and the use of HP as controls may affect the robustness of the results and raise the possibility of overfitting, despite additional validation using qPCR and iPRM.

Future studies with larger and more diverse cohorts, including comparisons between patients with and without BSS, are warranted to enhance statistical power, confirm the diagnostic utility of candidate biomarkers, and improve the generalizability of our findings. In addition, further basic experimental studies and validation across IHF subgroups using multiple TCM formulas would provide deeper insights into the mechanisms underlying different syndrome types and support the development of more precise therapeutic strategies. In addition, incorporating modern diagnostic tools, such as tongue and pulse imaging devices, will enable objective quantification of traditional TCM diagnostic methods and the collection of multimodal clinical data. Integrating these data with artificial intelligence and bioinformatics approaches can deepen our understanding of the relationships between clinical manifestations and underlying biological mechanisms, identify key therapeutic targets of TCM interventions, and ultimately facilitate the clinical translation of TCM while supporting more personalized treatment strategies for patients with IHF.

## 5 Conclusion

Complement and coagulation cascades, along with BCR signaling, play pivotal roles in the pathogenesis of BSS in IHF. A BSS-specific molecular interaction network was identified, involving key components such as F2, F8, F9, C3, FN1, CR2, CD19, CD22, CD79A, and CD79B. Among them, F2, F8, F9, and FN1 emerged as potential diagnostic and therapeutic targets. In addition, 5-hydroxyindoleacetic acid and 4-acetamidobutanoate were highlighted as candidate metabolic markers. These findings offer new insights into the biological basis of BSS in IHF and may inform future strategies for TCM-based diagnosis and therapy. However, further validation is required to confirm these conclusions.

## Data Availability

The raw sequence data reported in this paper have been deposited in the Genome Sequence Archive in National Genomics Data Center (Nucleic Acids Res 2022), China National Center for Bioinformation/Beijing Institute of Genomics, Chinese Academy of Sciences (GSA-Human: HRA011196) that are publicly accessible at: https://ngdc.cncb.ac.cn/gsa-human.

## Glossary

**6M** **WT**: 6-min walk test
APTT: activated partial thromboplastin time
BMI: body mass index
BSS: blood stasis syndrome
DBP: diastolic blood pressure
DEGs: differentially expressed genes
DEPs: differentially expressed proteins
DMs: differential metabolites
ELISA: enzyme-linked immunosorbent assay
FDR: false discovery rate
FIB: fibrinogen
GO: Gene Ontology
HDL-C: high-density lipoprotein cholesterol
HF: heart failure
HP: healthy participants
IHF: ischemic heart failure
iPRM: intelligent parallel reaction monitoring
KEGG: Kyoto Encyclopedia of Genes and Genomes
LVEF: left ventricular ejection fraction
MLHFQ: Minnesota Living with Heart Failure Questionnaire
NT-proBNP: N-terminal pro-B-type natriuretic peptide
PCA: principal component analysis
PPI: protein–protein interaction
PT: prothrombin time
QXXYZ: Qi deficiency with blood stasis syndrome
ROC: receiver operating characteristic
RT-qPCR: real-time quantitative polymerase chain reaction
TC: total cholesterol
TCM: traditional Chinese medicine
TT: thrombin time
YQHX: Yiqi Huoxue
YXXYSYZ: Yang deficiency with blood stasis accompanied by fluid retention syndrome
YXXYZ: Yang deficiency with blood stasis syndromeSupplementary Information.
