# Supplementary material for: Exploring molecular characteristics and interactions of blood stasis syndrome in ischemic heart failure by integrated multi-omics
Source: Front Mol Biosci. 2025 Oct 13;12:1627849. doi: 10.3389/fmolb.2025.1627849 (PMC12554561; doi:10.3389/fmolb.2025.1627849)
Supplement: Supplementary file 1 [file Supplementaryfile1.docx]

**Supplementary Material 1**

**Exploring Molecular Characteristics and Interactions of Blood Stasis Syndrome in Ischemic Heart Failure by integrated Multi-omics**

**S1. Data-independent-acquisition-based proteomic study**

**S1.1 Sample preparation**

Venous blood samples (2 mL) collected in EDTA-anticoagulated tubes from fasting subjects were centrifuged at 3,000×g for 15 min at 4°C. The supernatant was transferred to pre-labeled Eppendorf tubes, aliquoted, and stored at -80°C. Protein extraction and processing followed manufacturer protocols. High-abundance proteins were depleted using the ProteoMiner™ Low-Abundance Protein Enrichment Kit (Bio-Rad, USA). Protein concentration was quantified via Bradford assay (Biyuntian, China). The desalted peptide fragments were subjected to identification using liquid chromatography-tandem mass spectrometry (LC-MS/MS) (for detailed methodology, refer to Supplementary File S1). DEPs were identified using thresholds of upregulated proteins (FC > 1.2), downregulated proteins (FC < 0.83), and p < 0.05. Functional enrichment analysis of GO terms and KEGG pathways was performed using R package "Clusterprofiler" and "DOSE".

**S1.2 LC-MS/MS Analysis-DIA mode**

Prepare mobile phase A (100% water, 0.1% formic acid) and B (80% acetonitrile, 0.1% formic acid). The lyophilized powder was dissolved using 10µLA solution, centrifuged at 14,000g for 20min at 4°C, and 200 ng of the supernatant sample was injected into the sample for liquid-quality detection. The Vanquish Neo upgraded UHPLC system was used with a C18 pre-column of 174500 (5 mm×300 μ m,5 μm, Thermo Fisher，USA) heated at 50°C in a column oven, and a C18 analytical column of ES906 (PepMap TM Neo UHPLC 150µm x 15 cm, 2 μm, Thermo Fisher，USA). The Orbitrap Astral masss pectrometer was used (Thermo Fisher，USA), an ESI ion source was used, the ion spray voltage was set to 1.9 kV, the ion transfer tube temperature was set to 290°C, and the mass spectrum was in a data-dependent acquisition mode, with a full first-stage mass spectrometry scanning range of m/z 380-980. The primary MS resolution was set to 240000 (200m/z), AGC was set to 500%, the parent ion window size was set to 2-Th, the number of DIA windows was 300, the NCE was set to 25%, the secondary m/z acquisition range was from 150 to 2000, the sub-ion resolution Astral was set to 80000, and the maximal injection time was 3ms. Into mass spectrometry detection raw data (.raw).

**S1.3 Trusted protein analysis**

The raw files were searched and analyzed using the DIA-NN library search software, according to the homo_sapiens_uniprot_2023_10_18_Swissprot.fasta （20427 sequences） database. The library search parameters were set as follows: a mass tolerance of 10 ppm for precursor ions and 0.02 Da for fragment ions. Cysteine was modified by alkylation, methionine was oxidatively modified, and N-terminal modifications included acetylation, loss of methionine, and loss of methionine + acetylation. One missed cleavage site was allowed at most. To improve the quality of the analytical results, the DIA-NN software further filtered the search results by retaining only credible PSMs with a confidence level of 99% or higher. Only credible spectral peptides and proteins were retained, and FDR validation was performed to remove peptides and proteins with an FDR greater than 1%.

**S2. Targeted metabolomics analysis**

**S2.1 Sample preparation**

Fasting venous blood samples were collected from participants in the morning and aliquoted into 2 ml anticoagulant tubes containing EDTA for centrifugation. The supernatant was centrifuged at 4°C at 3000 ×g for 15 minutes, collected into Eppendorf tubes, labeled, and stored at -80°C. Target metabolite extraction was performed. A 100 μ L sample was combined with 300 μ L of methanol-water solution, vortexed, and left on ice for 15 minutes. The mixture was centrifuged at 12000 rpm at 4°C for 15 minutes. A 50 μL aliquot of the supernatant was mixed with 150 μL of derivatization reagent and incubated at 40°C for 40 minutes. Subsequently, 90 μL of the supernatant was combined with 10 μL of internal standard solution, vortexed, and subjected to LC-MS analysis. Accurate quantitative analysis was conducted for 478 metabolites, including 92 amino acids, 62 aromatic compounds, 43 organic acids, 38 bile acids, 19 fatty acids, 26 carbohydrates, 24 indoles, 23 nucleosides, nucleotides, and analogs, 14 phenylpropanoids, 9 pyridines, and 128 other compounds. The metabolites were sourced from Zhenzhun Biotechnology Co., Ltd. (Shanghai), Aladdin Biochemical Technology Co., Ltd. (Shanghai), and Sigma-Aldrich (USA).

**S2.2 Standard curve establishment**

Accurately weigh the standard substances of each metabolite, prepare a mixed stock solution, and dilute it with methanol to obtain a series of working solutions at different concentrations. Prepare an internal standard solution at a defined concentration, mix well, and obtain the final internal standard solution. The stock and working solutions of the linear standards, internal standards, and quality controls were stored at -20°C. Perform LC-MS analysis on the concentration series of standard solutions. The ratio of the standard concentration to the internal standard concentration is plotted on the x-axis, and the ratio of the peak area of the standard to that of the internal standard is plotted on the y-axis to evaluate the linearity of the standard solutions.

**S2.3 LC-MS/MS Analysis**

UHPLC-MS/MS system (ExionLC ™ AD UHPLC-QTRAP 6500+, AB SCIEX Corp., Boston, MA, USA) was used to quantitate metabolite. Separation was performed on a Waters HSS T3 column (2.1×150mm) which was maintained at 40°C. The mobile phase, consisting of 0.1% formic acid in water (solvent A) and acetonitrile/isopropanol (1:1) (solvent B), was delivered at a flow rate of 0.30 mL/min. The solvent gradient was set as follows: 5% B, 1 min; 5-40% B, 7 min; 40-95% B, 25 min; 95-5% B, 27.1 min; 30 min; 5% B. The mass spectrometer was operated in positive/negative multiple reaction mode (MRM) mode. Parameters were as follows: IonSpray Voltage (4500V/-4500V), Sheath Gas (35psi), Ion Source Temp (550°C), Auxiliary Gas (50psi), Collision Gas (55psi).

**S2.4 Data processing and analysis**

Metabolomics data were first processed using the metaX software, followed by dimensionality reduction and exploratory analysis through principal component analysis (PCA) and partial least squares discriminant analysis (PLS-DA). The PLS-DA model was validated using 7-fold cross-validation, and when the number of biological replicates (n) was ≤ 3, a k-fold cross-validation with k = 2n was applied. Model performance was evaluated using the parameters R² and Q² obtained from the validation, with values closer to 1 indicating greater stability and reliability. Variable importance in projection (VIP) scores were derived for each metabolite, and statistical significance between groups was assessed using independent samples t-tests. Differential metabolites (DMs) were selected based on the combined criteria of VIP > 1, t-test P < 0.05, and fold change thresholds (FC > 1.2 for upregulated metabolites, FC < 0.833 for downregulated metabolites).

**S3. Composition of the Yiqi Huoxue (YQHX) Formula**

YQHX consisted of seven natural medicinal ingredients: 60g of Astragali Radix (Huangqi, Gansu, China, Lot No. 176 231202), 15g of Paeoniae Radix Rubra (Chishao, Guangxi, China, Lot No. 231203), 12g of Chuanxiong Rhizoma (Chuangxiong, Sichuan, China, Lot No. 240101), 20g of Angelicae Sinensis Radix (Danggui, Gansu, China, Lot No. 231202), 12g of Lumbricus (Dilong, Gansu, China, Lot No. 231203), 12g of Carthami Flos (Honghua, Xinjiang, China, Lot No. 231201), and 12g of Persicae Semen (Taoren, Hebei, China, Lot No. 181 2312081). All herbal components were sourced from The First Affiliated Hospital of Henan University of Chinese Medicine and processed into granules using modern pharmaceutical techniques—including extraction, concentration, drying, granulation, and sealing—by Jiangyin Tianjiang Pharmaceutical Co., Ltd. (Approval No. 2204317), exclusively for clinical research.
