# Supplementary material for: Exploring molecular characteristics and interactions of blood stasis syndrome in ischemic heart failure by integrated multi-omics
Source: Front Mol Biosci. 2025 Oct 13;12:1627849. doi: 10.3389/fmolb.2025.1627849 (PMC12554561; doi:10.3389/fmolb.2025.1627849)
Supplement: Supplementary file 2 [file Supplementaryfile3.docx]

## Supporting figures


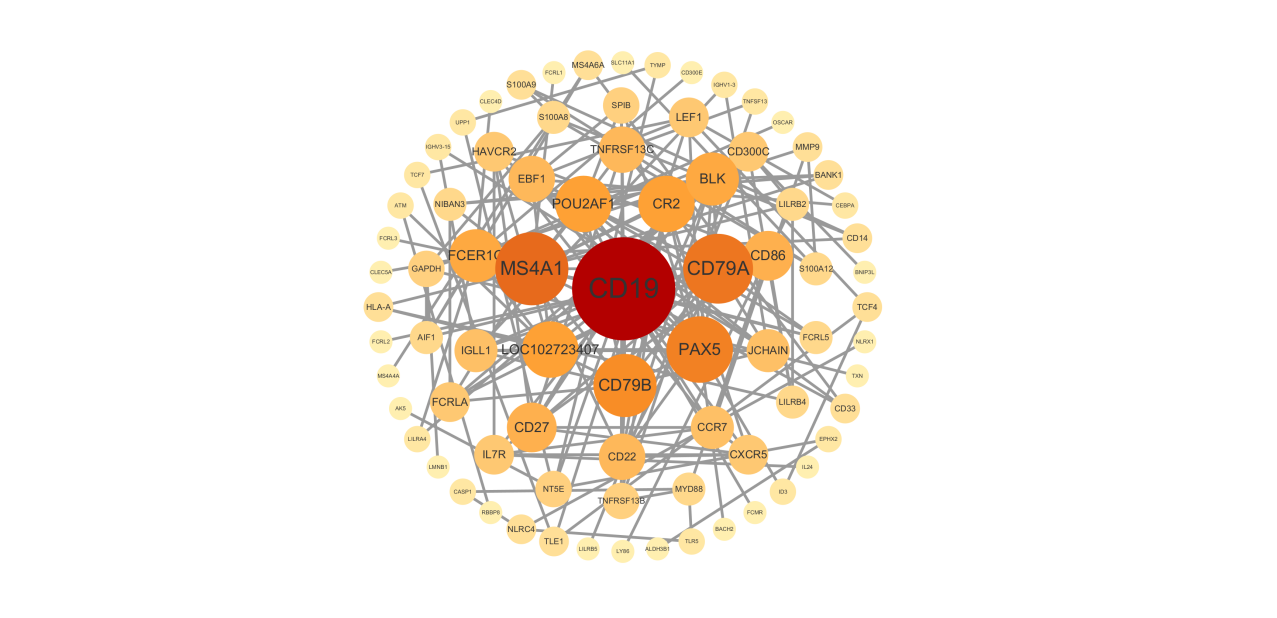


**Fig. S1.** Protein-protein interaction network of core differentially expressed genes


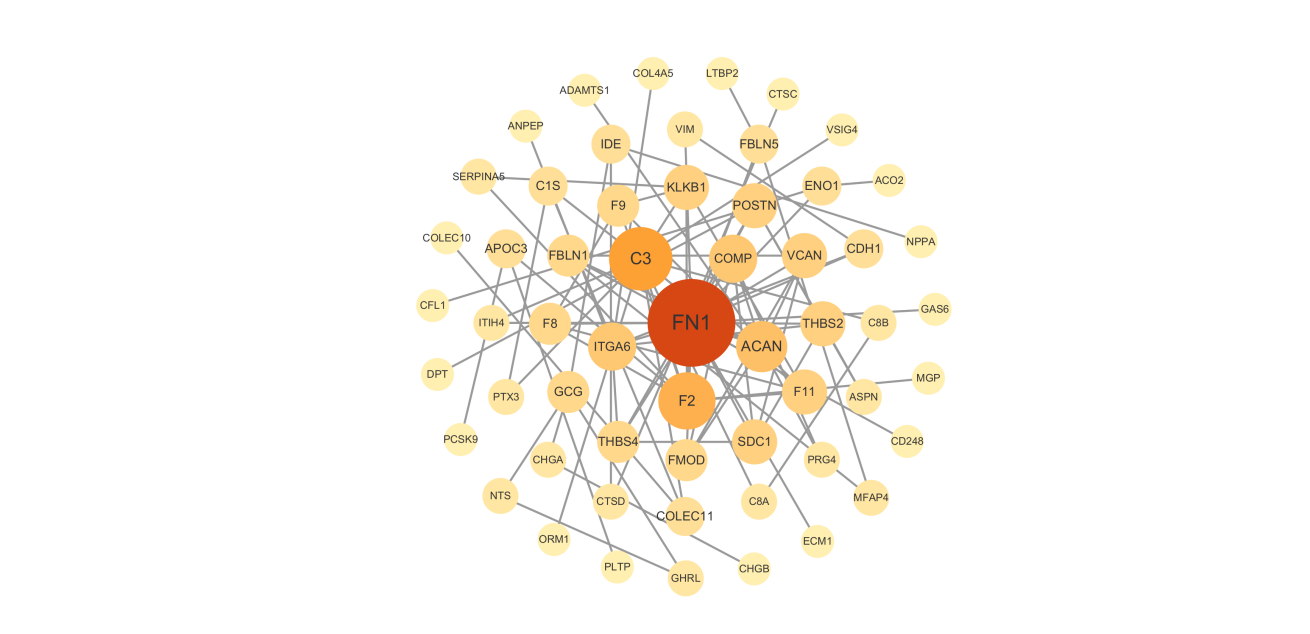


**Fig. S2.** Protein-protein interaction network of core differentially expressed proteins


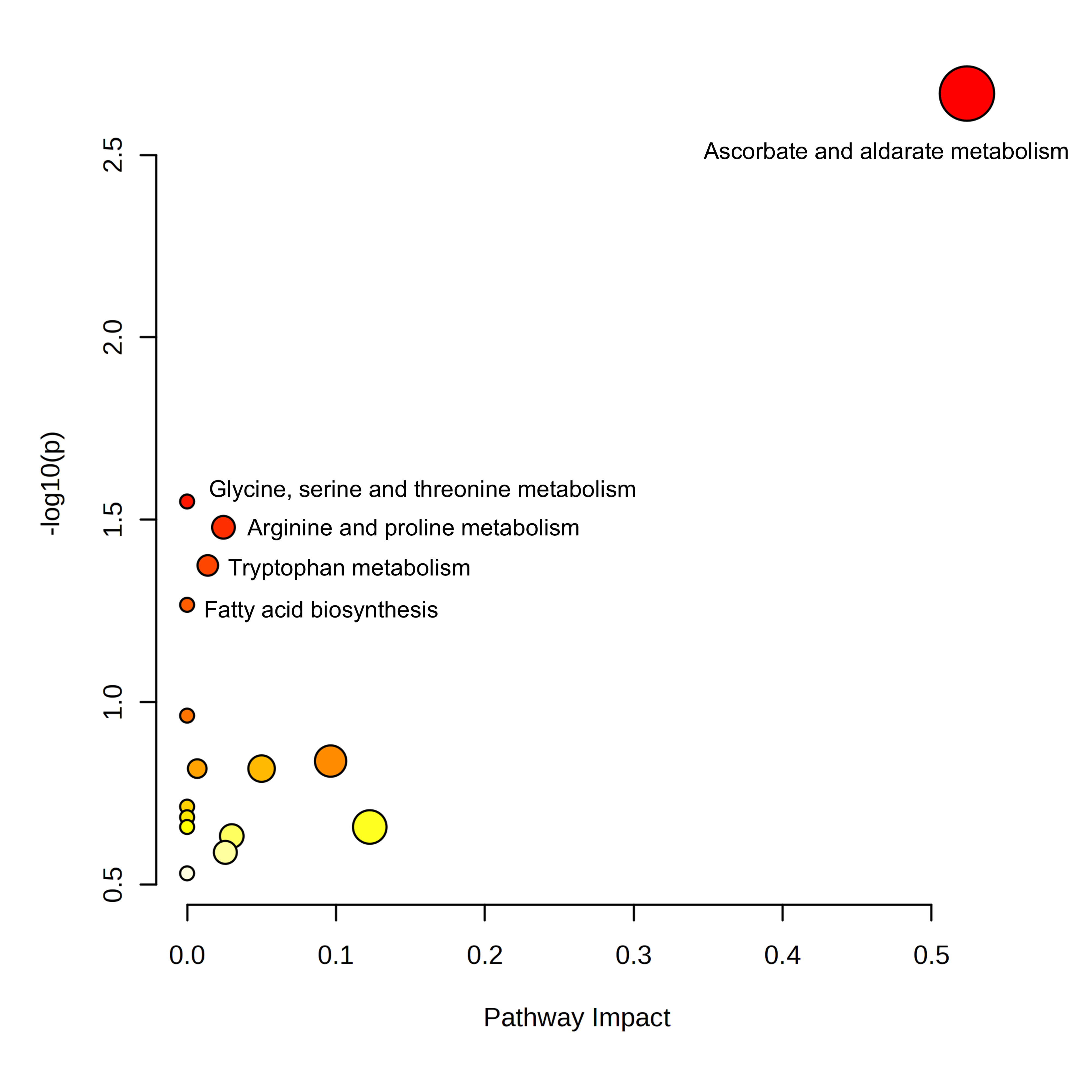


**Fig. S3.** Differential metabolic pathways of DMs enrichment in BSS Syndrome.

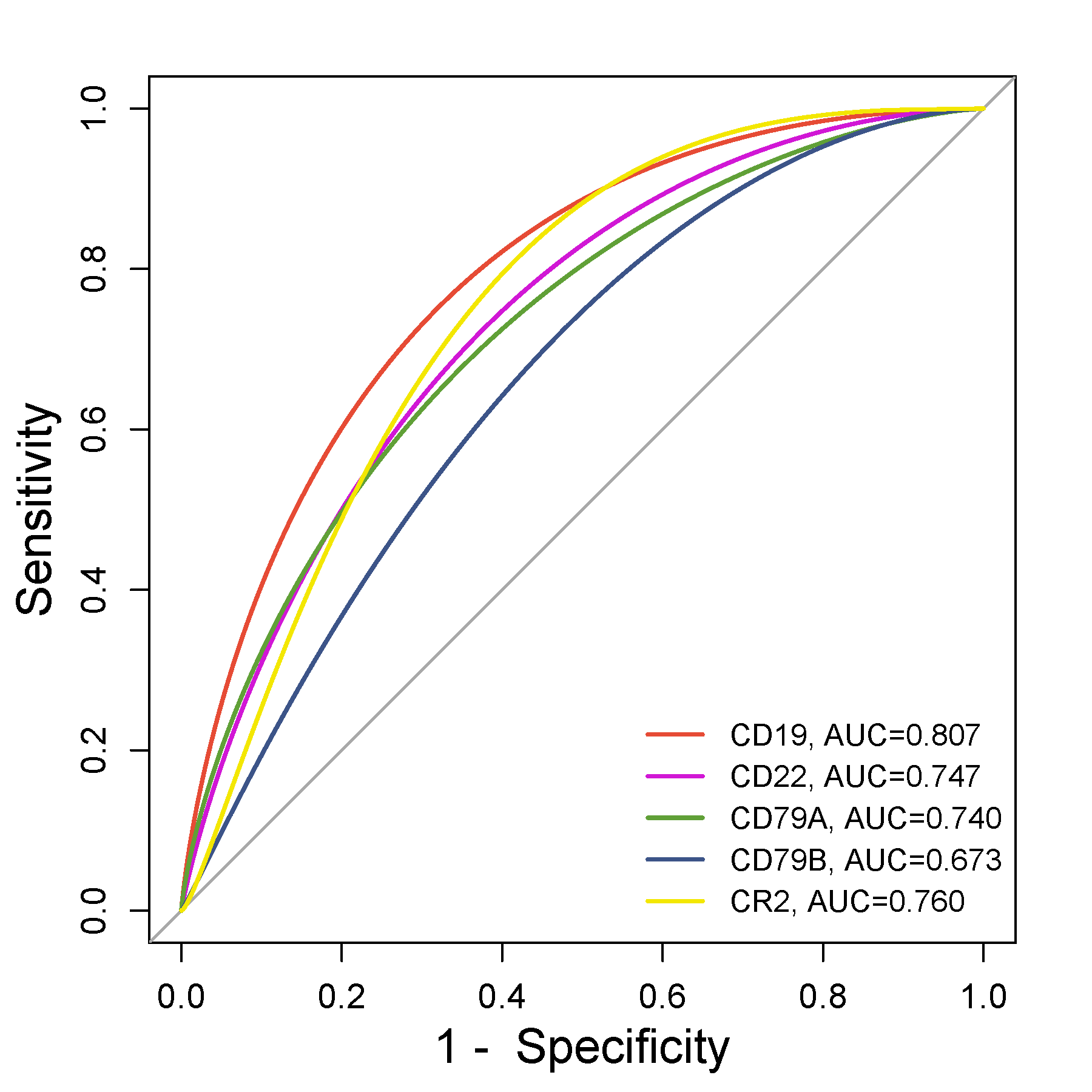


**Fig. S4.** ROC curves of hub genes for diagnostic prediction


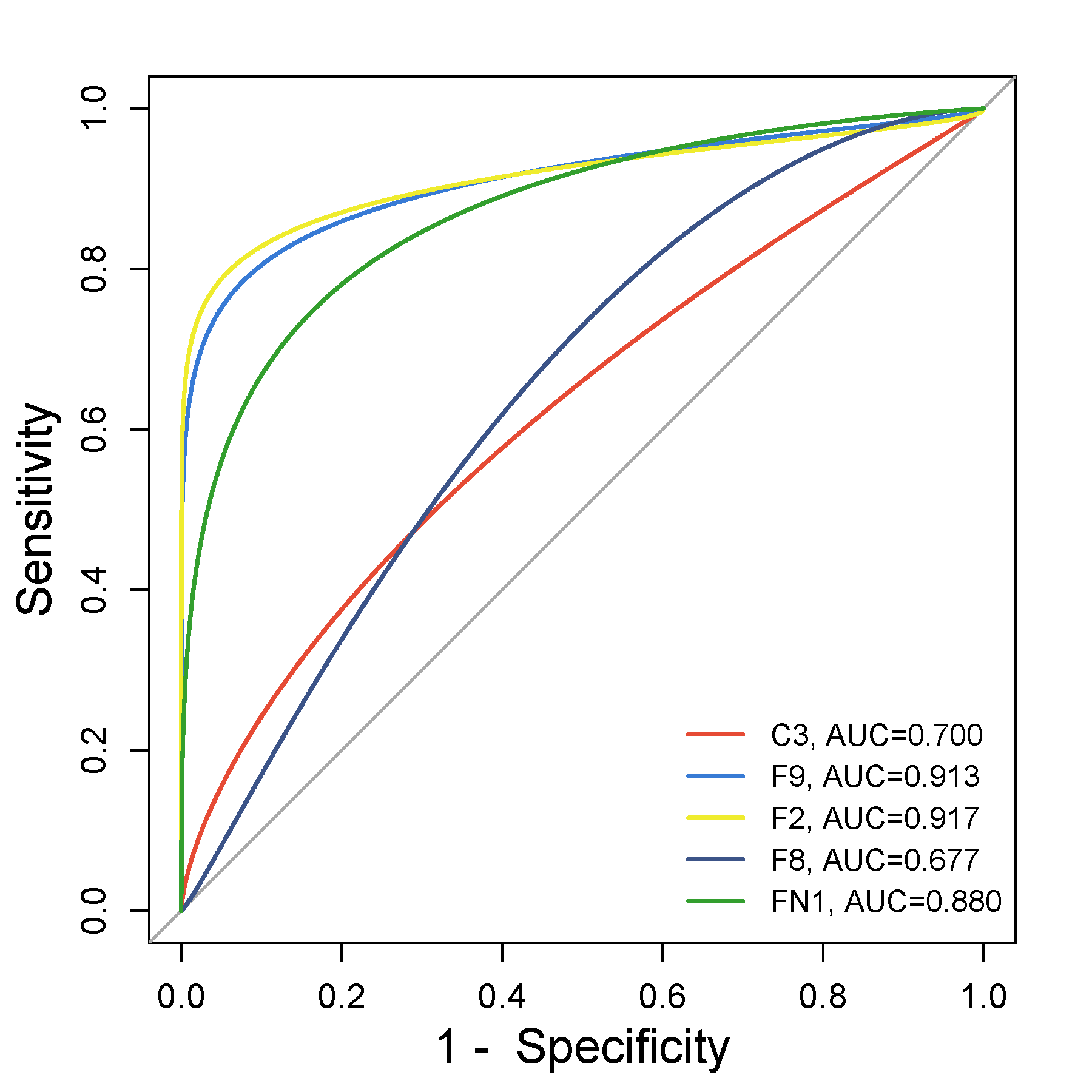

**Fig. S5.** ROC curves of hub proteins for diagnostic prediction


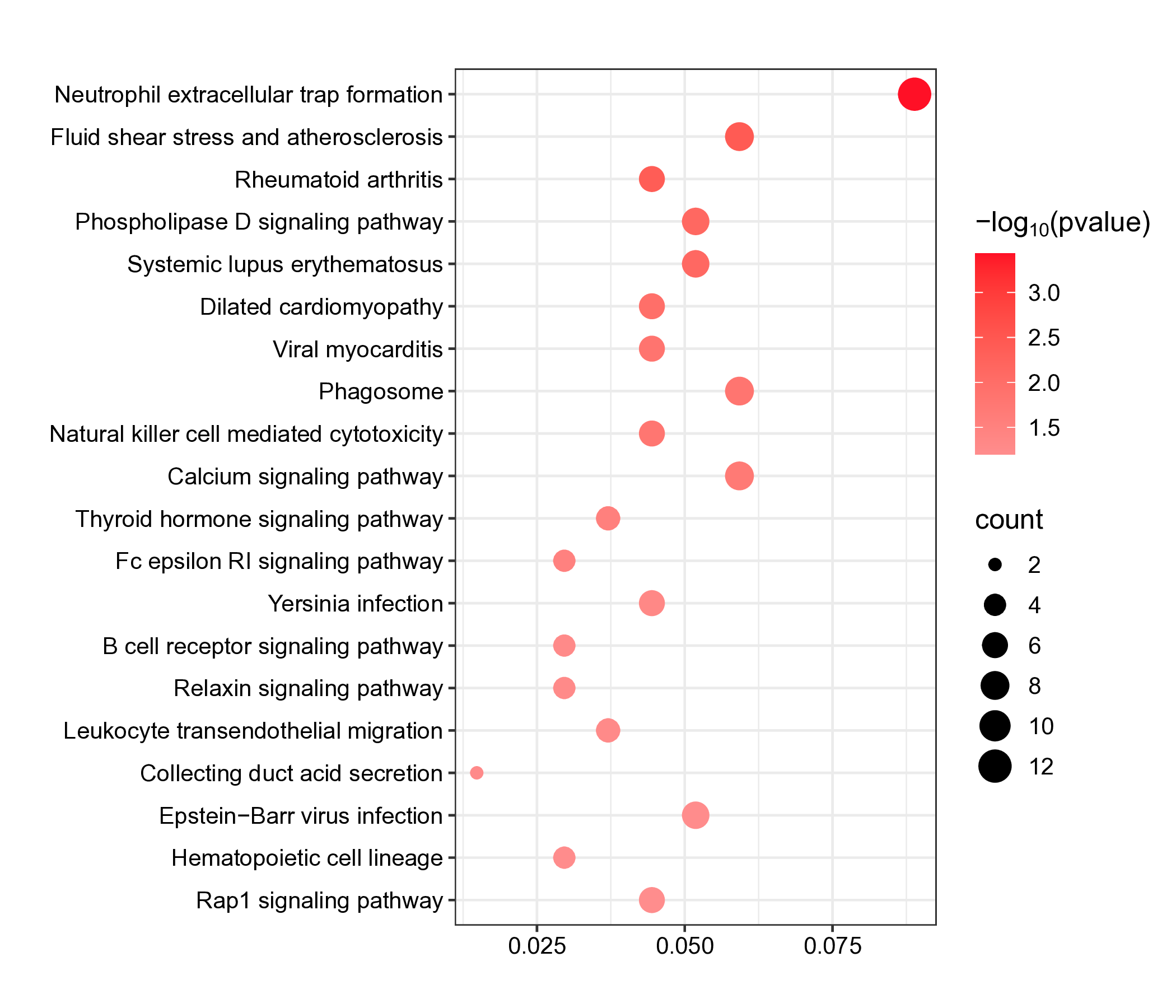


**Fig. S6.** KEGG Pathway Enrichment Analysis of Differentially Expressed Genes Before and After QYHX Treatment
