# Supplementary material for: Exploring molecular characteristics and interactions of blood stasis syndrome in ischemic heart failure by integrated multi-omics
Source: Front Mol Biosci. 2025 Oct 13;12:1627849. doi: 10.3389/fmolb.2025.1627849 (PMC12554561; doi:10.3389/fmolb.2025.1627849)
Supplement: Supplementary file 3 [file Supplementaryfile2.docx]

**Supplementary Material 1**

**Exploring Molecular Characteristics and Interactions of Blood Stasis Syndrome in Ischemic Heart Failure by integrated Multi-omics**

**Table S1. Diagnostic criteria for syndrome types**

| TCM syndrome differentiation strictly adhered to the Traditional Chinese Medicine Guideline for Diagnosis and Treatment of Chronic Heart Failure(2022) | |
| --- | --- |
| Qi deficiency and blood stasis Syndrome | Main symptoms: shortness of breath/ respite, fatigue, palpitation.  Secondary symptoms: (1) Tiredness and laziness, easy to exert oneself in activities; (2) Sweating; (3) low voice; (4) The complexion/lips are dark purple.  Tongue and pulse: Purple-dark tongue (or with ecchymosis, petechiae or sublingual varices), normal-sized tongue with white coating, and deep, thin or weak pulse. |
| Yang deficiency and blood stasis Syndrome | Main symptoms: shortness of breath/ respite, fatigue, palpitation.  Secondary symptoms: (1) Afraid of cold or like warm; (2) Stomach duct/abdomen/ waist/limbs feeling cold; (3) The body feels cold, accompanied by sweating; (4) The complexion/lips are dark purple.  Tongue and pulse: Purple-dark tongue (or with ecchymosis, petechiae or sublingual varices), fat tongue or teeth marks, thin, deep and slow/weak pulse. |
| Yang deficiency blood stasis with fluid retention | Main symptoms: shortness of breath/ respite, fatigue, palpitation.  Secondary symptoms: (1) Afraid of cold or like warm; (2) Sweating; (3) Stomach duct/abdomen/waist/limbs feeling cold; (4) Cough and phlegm; (5) Abdominal bloating; (6) Floating face or swollen limbs; (7) Difficult urination; (8) Duct fullness or vomiting; (9) Thirst does not desire to drink; (10) Dizziness; (11) The complexion/lips are dark purple.  Tongue and pulse: Lubricated or greasy tongue, smooth pulse. |
| The diagnosis can only be recognized if the above syndromes have 2 main symptoms and 2 secondary symptoms respectively, combined with the tongue and pulse. | |

**Table S2.** Diagnostic Scale for Blood Stasis Syndrome (BSS)

| Diagnostic item | score |
| --- | --- |
| Persistent chest pain | 4 |
| Dark purple tongue or petechial ecchymosis of tongue | 4 |
| The sublingual veins are purple | 3 |
| Dark purple complexion | 3 |
| The body has petechiae or ecchymosis | 3 |
| Numbness of the limbs | 2 |
| Lips are dark purple or dark red | 2 |
| Uneven pulse | 2 |

**Note:** A total score ≥ 8 indicates the diagnosis of BSS.

**Table S3. Primer information**

| **Gene** |  | **Prinmer** | **Gene ID** | **Amplicon Size(bp)** |
| --- | --- | --- | --- | --- |
| hCD19-F | Forward | GGCCCGAGGAACCTCTAGT | 930 | 131 |
| hCD19-R | Reverse | TAAGAAGGGTTTAAGCGGGGA |  |  |
| hCD22-F | Forward | GCACCCTGAAACCCTCTACG | 933 | 136 |
| hCD22-R | Reverse | ATCAAACTTCGAGGTGTTCTTGT |  |  |
| hCR2-F | Forward | GGTCCTCGGGATTTCTTGTGG | 1380 | 105 |
| hCR2-R | Reverse | GAACAACTGTACCTTATCACGGT |  |  |
| hCD79A-F | Forward | CAAGAACCGAATCATCACAGCC | 973 | 92 |
| hCD79A-R | Reverse | TCTGCCATCGTTTCCTGAACA |  |  |
| hCD79B-F | Forward | AGGGCCTGGACATTGACCA | 974 | 81 |
| hCD79B-R | Reverse | CACCTACAGACCACTTCACTTC |  |  |

**Table S4. Comorbidities and Medication Usage in Patients with Different TCM Syndromes of Ischemic Heart Failure**

| Variable | QXXYZ  （n=30） | YXXYZ  （n=30） | YXXYSYZ  （n=30） | *p* value |
| --- | --- | --- | --- | --- |
| Hypertension | 12 (40.00%) | 13 (43.33%) | 14 (46.67%) | 0.873 |
| Diabetes Mellitus | 5 (16.67%) | 8 (26.67%) | 9 (30.00%) | 0.457 |
| Stroke | 5 (16.67%) | 6 (20.00%) | 4 (13.33%) | 0.787 |
| Hyperlipidemia | 6 (20.00%) | 4 (13.33%) | 3 (10.00%) | 0.538 |
| Atrial Fibrillation | 1 (3.33%) | 2 (6.67%) | 0 (0.00%) | 0.242 |
| EC-ASA | 20 (66.67%) | 14 (46.67%) | 16 (53.33%) | 0.284 |
| Clopidogrel | 9 (30.00%) | 13 (43.33%) | 17 (56.67%) | 0.114 |
| Statins | 26 (86.67%) | 23 (76.67%) | 24 (80.00%) | 0.602 |
| CCB | 5 (16.67%) | 1 (3.33%) | 2 (6.67%) | 0.174 |
| ACEI/ARB | 7 (23.33%) | 8 (26.67%) | 5 (16.67%) | 0.638 |
| ARNI | 9 (30.00%) | 13 (43.33%) | 12 (40.00%) | 0.541 |
| MRAs | 9 (30.00%) | 17 (56.67%) | 17 (56.67%) | 0.058 |
| SGLT2 | 2 (6.67%) | 7 (23.33%) | 3 (10.00%) | 0.143 |
| BB | 23 (76.67%) | 19 (63.33%) | 21 (70.00%) | 0.511 |

Data are presented as n (%). p values were calculated using the χ2 test or Fisher’s exact test. EC-ASA, enteric-coated acetylsalicylic acid; CCB, calcium channel blocker; ACEI/ARB, angiotensin-converting enzyme inhibitor/angiotensin receptor blocker; ARNI, angiotensin receptor–neprilysin inhibitor; MRAs, mineralocorticoid receptor antagonists; SGLT2, sodium-glucose co-transporter 2 inhibitor; BB, β-blocker

**Table S5. QXXYZ-associated differential metabolites**

| **Name** | **HMDB** | **Class** | **KEGG** |
| --- | --- | --- | --- |
| 3-Hydroxy-3-methylglutarate | HMDB0000355 | Fatty Acids | C03761 |
| Salicyluric acid | HMDB0000840 | Benzenoids | C07588 |
| Salicylic acid | HMDB0001895 | Benzenoids | C00805 |
| Adipic acid | HMDB0000448 | Fatty Acids | C06104 |
| 3,4-Dihydroxybutyrate | HMDB0000337 | Fatty Acids | NA |
| Hendecanoic acid | HMDB0000947 | Fatty Acids | C17715 |
| Creatine | HMDB0000064 | Amino Acids | C00300 |
| D-Glucuronic acid | HMDB0000127 | Carbohydrates | C00191 |
| D-Xylonic acid | HMDB0059750 | Carbohydrates | C00502 |
| Suberic acid | HMDB0000893 | Fatty Acids | C08278 |
| 5-Hydroxyindolacetic acid | HMDB0000763 | Indoles | C05635 |
| Malonic acid | HMDB0000691 | Organic Acids | C04025 |
| cis-Aconitic acid | HMDB0000072 | Organic Acids | C00417 |
| Methylsuccinic acid | HMDB0001844 | Fatty Acids | NA |
| Choline | HMDB0000097 | Cholines | C00114 |
| D-Gluconic Acid | HMDB0000625 | Carbohydrates | C00257 |
| (E)-3-Methylglutaconic acid | HMDB0000522 | Fatty Acids | NA |
| Caproic acid | HMDB0000535 | Short Chain Fatty Acids | C01585 |
| Sebacic acid | HMDB0000792 | Fatty Acids | C08277 |
| Valeric acid | HMDB0000892 | Short Chain Fatty Acids | C00803 |
| 2-Hydroxy-2-methylbutyric acid | HMDB0001987 | Organic Acids | NA |
| (R)-3-(4-Hydroxyphenyl)lactate | HMDB0000755 | Phenylpropionic Acids | C03964 |
| 3-Methylpentanoic acid | HMDB0033774 | Short Chain Fatty Acids | NA |
| Nonanoic acid | HMDB0000847 | Fatty Acids | C01601 |
| Alpha-N-Phenylacetyl-L-glutamine | HMDB0006344 | Amino Acids | C04148 |
| L-Acetylcarnitine | HMDB0000201 | Carnitines | C02571 |
| Linoleic acid amide | HMDB0062656 | Fatty Amides | NA |
| Phenyllactic acid | HMDB0000779 | Phenylpropionic Acids | C05607 |
| Homocitrulline | HMDB0000679 | Amino Acids | C02427 |
| Quinolinic Acid | HMDB0000232 | Pyridines | C03722 |
| Azelaic acid | HMDB0000784 | Fatty Acids | C08261 |
| 5-Methyluridine | HMDB0000884 | Nucleosides, nucleotides, and analogues | NA |
| 2,6-Pyridinedicarboxylic acid | HMDB0033161 | Pyridines | NA |
| Taurolithocholic acid | HMDB0000722 | Bile Acids | C02592 |
| 2-Piperidinone | HMDB0011749 | Piperidines | NA |
| Caprylic acid | HMDB0000482 | Fatty Acids | C06423 |
| Tetracosanoic acid | HMDB0002003 | Fatty Acids | C08320 |
| 3-(2-Hydroxyphenyl)propanoic acid | HMDB0033752 | Phenylpropionic Acids | C01198 |
| 3β -Ursodeoxycholic acid | HMDB0000686 | Bile Acids | C17662 |
| Maleic acid | HMDB0000176 | Organic Acids | C01384 |
| 12-Ketolithocholic acid | HMDB0000328 | Bile Acids | NA |
| Acetaminophen | HMDB0001859 | Benzenoids | C06804 |
| L-(+)-Arabinose | HMDB0000646 | Carbohydrates | C02479 |
| D-Fructose | HMDB0000660 | Carbohydrates | C00095 |
| 3-Hydroxypicolinic acid | HMDB0013188 | Pyridines | C18620 |
| 3-Indoleglyoxylic Acid | HMDB0242143 | Indoles | NA |
| L-Histidine | HMDB0000177 | Amino Acids | C00135 |
| Dodecanedioic acid | HMDB0000623 | Fatty Acids | C02678 |
| Gulonolactone | HMDB0003466 | Lactones | C01040 |
| Ursodeoxycholic acid | HMDB0000946 | Bile Acids | C07880 |
| 4-Acetamidobutanoate | HMDB0003681 | Amino Acids | C02946 |
| Lithocholic acid | HMDB0000761 | Bile Acids | C03990 |
| Benzoic acid | HMDB0001870 | Benzenoids | C00180 |
| 3-Methoxyanthranilate | HMDB0060374 | Benzenoids | C05831 |
| Maleamic acid | NA | Organic Acids | C01596 |
| 2-Keto-D-Gluconic acid | HMDB0011732 | Carbohydrates | C06473 |
| Decanoic acid | HMDB0000511 | Fatty Acids | C01571 |
| Oxoadipic acid | HMDB0000225 | Organic Acids | C00322 |
| p-Hydroxyphenylacetic acid | HMDB0000020 | Benzenoids | C00642 |
| Adenosine | HMDB0000050 | Nucleosides, nucleotides, and analogues | C00212 |
| Alpha-Hydroxyisobutyric acid | HMDB0000729 | Organic Acids | C21297 |
| Docosanoic acid | HMDB0000944 | Fatty Acids | C08281 |
| N6-Acetyllysine | HMDB0000206 | Amino Acids | C02727 |
| N-Acetyl-L-glutamic acid | HMDB0001138 | Amino Acids | C00624 |
| But-2-enoic acid | HMDB0010720 | Fatty Acids | C01771 |
| Threonic acid | HMDB0000943 | Organic Acids | C01620 |
| 3-Methyladipic acid | HMDB0000555 | Fatty Acids | NA |
| Hydantoin-5-propionate | HMDB0001212 | Organoheterocyclic compounds | C05565 |
| 4-Methylbenzoic acid | HMDB0029635 | Benzenoids | C01454 |
| L-Threonine | HMDB0000167 | Amino Acids | C00188 |
| 3-Hydroxyisovaleric acid | HMDB0000754 | Organic Acids | C20827 |
| 3-Methyl-2-oxovaleric acid | HMDB0000491 | Organic Acids | C00671 |
| Methylglutaric acid | HMDB0000752 | Fatty Acids | NA |
| Pantothenic acid | HMDB0000210 | Alcohols | C00864 |
| Lactose | HMDB0000186 | Carbohydrates | C00243 |

**Table S6. YXXYZ-associated differential metabolites**

| **Name** | **HMDB** | **Class** | **KEGG** |
| --- | --- | --- | --- |
| 3-Hydroxy-3-methylglutarate | HMDB0000355 | Fatty Acids | C03761 |
| D-Glucuronic acid | HMDB0000127 | Carbohydrates | C00191 |
| (E)-3-Methylglutaconic acid | HMDB0000522 | Fatty Acids | NA |
| 3,4-Dihydroxybutyrate | HMDB0000337 | Fatty Acids | NA |
| Salicyluric acid | HMDB0000840 | Benzenoids | C07588 |
| Homocitrulline | HMDB0000679 | Amino Acids | C02427 |
| Methylglutaric acid | HMDB0000752 | Fatty Acids | NA |
| Choline | HMDB0000097 | Cholines | C00114 |
| Salicylic acid | HMDB0001895 | Benzenoids | C00805 |
| 5-Hydroxyindolacetic acid | HMDB0000763 | Indoles | C05635 |
| N-Acetylserine | HMDB0002931 | Amino Acids | NA |
| Caprylic acid | HMDB0000482 | Fatty Acids | C06423 |
| Adipic acid | HMDB0000448 | Fatty Acids | C06104 |
| Decanoic acid | HMDB0000511 | Fatty Acids | C01571 |
| Sebacic acid | HMDB0000792 | Fatty Acids | C08277 |
| (R)-3-(4-Hydroxyphenyl)lactate | HMDB0000755 | Phenylpropionic Acids | C03964 |
| 4-Acetamidobutanoate | HMDB0003681 | Amino Acids | C02946 |
| Suberic acid | HMDB0000893 | Fatty Acids | C08278 |
| Creatine | HMDB0000064 | Amino Acids | C00300 |
| Hendecanoic acid | HMDB0000947 | Fatty Acids | C17715 |
| 3-Hydroxydecanoate | HMDB0002203 | Fatty Acids | NA |
| D-Xylonic acid | HMDB0059750 | Carbohydrates | C00502 |
| Alpha-N-Phenylacetyl-L-glutamine | HMDB0006344 | Amino Acids | C04148 |
| cis-Aconitic acid | HMDB0000072 | Organic Acids | C00417 |
| Alpha-Hydroxyisobutyric acid | HMDB0000729 | Organic Acids | C21297 |
| 3-Hydroxybutyrate | HMDB0000011 | Fatty Acids | C01089 |
| Mevalonic acid | HMDB0000227 | Fatty Acids | C00418 |
| Pantothenic acid | HMDB0000210 | Alcohols | C00864 |
| Oxoadipic acid | HMDB0000225 | Organic Acids | C00322 |
| Hydantoin-5-propionate | HMDB0001212 | Organoheterocyclic compounds | C05565 |
| N-Acetyl-L-glutamic acid | HMDB0001138 | Amino Acids | C00624 |
| Dodecanedioic acid | HMDB0000623 | Fatty Acids | C02678 |
| L-Lactic acid | HMDB0000190 | Organic Acids | C00186 |
| Methylsuccinic acid | HMDB0001844 | Fatty Acids | NA |
| Kynurenic acid | HMDB0000715 | Quinolines | C01717 |
| trans-10-Heptadecenoic acid | HMDB0244268 | Fatty Acids | NA |
| Palmitelaidic acid | HMDB0012328 | Fatty Acids | NA |
| 3-Hydroxylaurate | HMDB0000387 | Fatty Acids | NA |
| N-Acetylcitrulline | HMDB0000856 | Amino Acids | C15532 |
| N6-Acetyllysine | HMDB0000206 | Amino Acids | C02727 |
| Phenyllactic acid | HMDB0000779 | Phenylpropionic Acids | C05607 |
| 3-Methyladipic acid | HMDB0000555 | Fatty Acids | NA |
| Pyrrole-2-carboxylic acid | HMDB0004230 | Pyrroles | C05942 |
| cis-7,10,13,16-Docosic acidtraenoic acid | HMDB0002226 | Fatty Acids | C16527 |
| Gamma-Glutamylglutamate | HMDB0011737 | Amino Acids | C05282 |
| Imidazolepropionic acid | HMDB0002271 | Imidazoles | C20522 |
| Ricinoleic acid | HMDB0034297 | Fatty Acids | C08365 |
| N-Acetylphenylalanine | HMDB0000512 | Amino Acids | C03519 |
| N-Acetylglutamine | HMDB0006029 | Amino Acids | NA |
| Tetradecanoic acid | HMDB0000806 | Fatty Acids | C06424 |
| N-Acetyl-L-alanine | HMDB0000766 | Amino Acids | NA |
| Fumaric Acid | HMDB0000134 | Organic Acids | C00122 |
| 3β -Ursodeoxycholic acid | HMDB0000686 | Bile Acids | C17662 |
| 2-Hydroxy-2-methylbutyric acid | HMDB0001987 | Organic Acids | NA |
| N-Alpha-acetyllysine | HMDB0000446 | Amino Acids | C12989 |
| L-Threonine | HMDB0000167 | Amino Acids | C00188 |
| Maleic acid | HMDB0000176 | Organic Acids | C01384 |
| 7,8-Dihydroneopterin | HMDB0002275 | Pteridines | C04874 |
| L-Fucose | HMDB0000174 | Carbohydrates | C01019 |
| Hexadecanoic acid | HMDB0000220 | Fatty Acids | C00249 |
| L-Alanine | HMDB0000161 | Amino Acids | C00041 |
| Xanthurenic acid | HMDB0000881 | Quinolines | C02470 |
| 2,6-Pyridinedicarboxylic acid | HMDB0033161 | Pyridines | NA |
| Gulonolactone | HMDB0003466 | Lactones | C01040 |
| 2-Piperidinone | HMDB0011749 | Piperidines | NA |
| 2,2-Dimethylsuccinic acid | HMDB0002074 | Fatty Acids | NA |
| 1-Methylhistidine | HMDB0000001 | Amino Acids | C01152 |
| Glucaric acid | HMDB0000663 | Carbohydrates | C00818 |
| L-Cysteinesulfinic acid | HMDB0060179 | Amino Acids | C00606 |
| 2-Hydroxybutyric acid | HMDB0000008 | Fatty Acids | C05984 |
| Heptadecanoic acid | HMDB0002259 | Fatty Acids | NA |
| Itaconic acid | HMDB0002092 | Organic Acids | C00490 |
| 3-Hydroxypicolinic acid | HMDB0013188 | Pyridines | C18620 |
| p-Hydroxyphenylacetic acid | HMDB0000020 | Benzenoids | C00642 |
| 3-Hydroxyisovaleric acid | HMDB0000754 | Organic Acids | C20827 |
| Phenylalanylvaline | HMDB0029008 | Amino Acids | NA |

**Table S7. YXXYSYZ-associated differential metabolites**

| **Name** | **HMDB** | **Class** | **KEGG** |
| --- | --- | --- | --- |
| 3-Hydroxy-3-methylglutarate | HMDB0000355 | Fatty Acids | C03761 |
| 3,4-Dihydroxybutyrate | HMDB0000337 | Fatty Acids | NA |
| D-Xylonic acid | HMDB0059750 | Carbohydrates | C00502 |
| 3-Indoleglyoxylic Acid | HMDB0242143 | Indoles | NA |
| Choline | HMDB0000097 | Cholines | C00114 |
| (R)-3-(4-Hydroxyphenyl)lactate | HMDB0000755 | Phenylpropionic Acids | C03964 |
| cis-Aconitic acid | HMDB0000072 | Organic Acids | C00417 |
| D-Glucuronic acid | HMDB0000127 | Carbohydrates | C00191 |
| Alpha-Hydroxyisobutyric acid | HMDB0000729 | Organic Acids | C21297 |
| Salicyluric acid | HMDB0000840 | Benzenoids | C07588 |
| Creatine | HMDB0000064 | Amino Acids | C00300 |
| Hydantoin-5-propionate | HMDB0001212 | Organoheterocyclic compounds | C05565 |
| 5-Hydroxyindolacetic acid | HMDB0000763 | Indoles | C05635 |
| Sebacic acid | HMDB0000792 | Fatty Acids | C08277 |
| Alpha-N-Phenylacetyl-L-glutamine | HMDB0006344 | Amino Acids | C04148 |
| Gulonolactone | HMDB0003466 | Lactones | C01040 |
| N-Acetylcitrulline | HMDB0000856 | Amino Acids | C15532 |
| 2-Piperidinone | HMDB0011749 | Piperidines | NA |
| Methylsuccinic acid | HMDB0001844 | Fatty Acids | NA |
| Hendecanoic acid | HMDB0000947 | Fatty Acids | C17715 |
| N6-Acetyllysine | HMDB0000206 | Amino Acids | C02727 |
| Phenyllactic acid | HMDB0000779 | Phenylpropionic Acids | C05607 |
| 2-Hydroxy-2-methylbutyric acid | HMDB0001987 | Organic Acids | NA |
| Homocitrulline | HMDB0000679 | Amino Acids | C02427 |
| N-Acetyl-L-glutamic acid | HMDB0001138 | Amino Acids | C00624 |
| Pantothenic acid | HMDB0000210 | Alcohols | C00864 |
| Lithocholic acid | HMDB0000761 | Bile Acids | C03990 |
| Dodecanedioic acid | HMDB0000623 | Fatty Acids | C02678 |
| N-Acetylglutamine | HMDB0006029 | Amino Acids | NA |
| N-Acetylserine | HMDB0002931 | Amino Acids | NA |
| Quinolinic Acid | HMDB0000232 | Pyridines | C03722 |
| 4-Acetamidobutanoate | HMDB0003681 | Amino Acids | C02946 |
| D-Gluconic Acid | HMDB0000625 | Carbohydrates | C00257 |
| Salicylic acid | HMDB0001895 | Benzenoids | C00805 |
| Caprylic acid | HMDB0000482 | Fatty Acids | C06423 |
| (E)-3-Methylglutaconic acid | HMDB0000522 | Fatty Acids | NA |
| N-Acetyl-L-alanine | HMDB0000766 | Amino Acids | NA |
| Valeric acid | HMDB0000892 | Short Chain Fatty Acids | C00803 |
| Nonanoic acid | HMDB0000847 | Fatty Acids | C01601 |
| p-Hydroxyphenylacetic acid | HMDB0000020 | Benzenoids | C00642 |
| Malonic acid | HMDB0000691 | Organic Acids | C04025 |
| Caproic acid | HMDB0000535 | Short Chain Fatty Acids | C01585 |
| Suberic acid | HMDB0000893 | Fatty Acids | C08278 |
| N-Acetyl-L-tryptophan | HMDB0013713 | Amino Acids | NA |
| Maleic acid | HMDB0000176 | Organic Acids | C01384 |
| N-Acetylphenylalanine | HMDB0000512 | Amino Acids | C03519 |
| Mevalonic acid | HMDB0000227 | Fatty Acids | C00418 |
| 1-Methylinosine | HMDB0002721 | Nucleosides, nucleotides, and analogues | NA |
| Xanthurenic acid | HMDB0000881 | Quinolines | C02470 |
| Androsterone glucuronide | HMDB0002829 | Steroids | C11135 |
| 1-Methylhistidine | HMDB0000001 | Amino Acids | C01152 |
| Kynurenic acid | HMDB0000715 | Quinolines | C01717 |
| Phenylpyruvic acid | HMDB0000205 | Benzenoids | C00166 |
| L-(+)-Arabinose | HMDB0000646 | Carbohydrates | C02479 |
| Azelaic acid | HMDB0000784 | Fatty Acids | C08261 |
| L-Fucose | HMDB0000174 | Carbohydrates | C01019 |
| D-Fructose | HMDB0000660 | Carbohydrates | C00095 |
| Itaconic acid | HMDB0002092 | Organic Acids | C00490 |
| Decanoic acid | HMDB0000511 | Fatty Acids | C01571 |
| Oxoadipic acid | HMDB0000225 | Organic Acids | C00322 |
| Sphingosine | HMDB0000252 | Sphingolipids | C00319 |
| Glucaric acid | HMDB0000663 | Carbohydrates | C00818 |
| D-2-Hydroxyglutaric acid | HMDB0000606 | Organic Acids | C01087 |
| Phthalic acid | HMDB0002107 | Benzenoids | C01606 |
| Adipic acid | HMDB0000448 | Fatty Acids | C06104 |
| 3β -Ursodeoxycholic acid | HMDB0000686 | Bile Acids | C17662 |
| Glycolic acid | HMDB0000115 | Organic Acids | C00160 |
| But-2-enoic acid | HMDB0010720 | Fatty Acids | C01771 |
| Trimethylgallic acid | HMDB0033839 | Benzenoids | NA |
| 2,6-Pyridinedicarboxylic acid | HMDB0033161 | Pyridines | NA |
| 3-Hydroxypicolinic acid | HMDB0013188 | Pyridines | C18620 |
| 3-Methyladipic acid | HMDB0000555 | Fatty Acids | NA |
| Methylglutaric acid | HMDB0000752 | Fatty Acids | NA |
| Oxamic acid | NA | Organic Acids | C01444 |
| Maleamic acid | NA | Organic Acids | C01596 |
| Tridecanoic acid | HMDB0000910 | Fatty Acids | C17076 |
| Hippuric acid | HMDB0000714 | Benzenoids | C01586 |
| Pyrrole-2-carboxylic acid | HMDB0004230 | Pyrroles | C05942 |
| 4-Methylbenzoic acid | HMDB0029635 | Benzenoids | C01454 |
| 4-Hydroxyproline | HMDB0000725 | Amino Acids | C01157 |
| Glycodeoxycholic acid | HMDB0000631 | Bile Acids | C05464 |
| 3-Methoxyanthranilate | HMDB0060374 | Benzenoids | C05831 |
| Tricarballylic acid | HMDB0031193 | Organic Acids | C19806 |
| Ursodeoxycholic acid | HMDB0000946 | Bile Acids | C07880 |
| gamma-Glutamylphenylalanine | HMDB0000594 | Amino Acids | NA |
| Elaidic acid(C18-1T) | HMDB0000573 | Fatty Acids | C01712 |
| Isolithocholic acid | HMDB0000717 | Bile Acids | C17658 |
| Taurohyodeoxycholic acid | NA | Bile Acids | NA |
| 4-Hydroxyhippuric acid | HMDB0013678 | Benzenoids | NA |
| 3-(2-Hydroxyphenyl)propanoic acid | HMDB0033752 | Phenylpropionic Acids | C01198 |
| Lactose | HMDB0000186 | Carbohydrates | C00243 |
| Tryptophol | HMDB0003447 | Indoles | C00955 |
| N-Acetylhistidine | HMDB0032055 | Amino Acids | C02997 |
| Taurolithocholic acid | HMDB0000722 | Bile Acids | C02592 |
| Ricinoleic acid | HMDB0034297 | Fatty Acids | C08365 |

**Table S8. Differential metabolites linked to BSS-centric proteins**

| Metabolite | F8 | F2 | F9 | C3 | FN1 |
| --- | --- | --- | --- | --- | --- |
| 3,4-Dihydroxybutyrate | 0.062 | -0.007 | 0.219* | -0.132 | 0.032 |
| 2-Piperidinone | -0.289** | -0.061 | -0.018 | 0.175 | -0.099 |
| Oxoadipic acid | -0.021 | 0.04 | 0.245* | -0.011 | -0.055 |
| 3β -Ursodeoxycholic acid | -0.143 | -0.04 | 0.014 | 0.041 | 0.032 |
| 4-Acetamidobutanoate | 0.021 | 0.037 | 0.236* | -0.179 | 0.007 |
| 5-Hydroxyindolacetic acid | 0.225* | 0.084 | 0.126 | -0.186 | -0.02 |
| D-Xylonic acid | 0.188 | 0.17 | 0.331** | -0.207 | 0.055 |
| Alpha-N-Phenylacetyl-L-glutamine | 0.11 | 0.085 | 0.169 | -0.267* | -0.004 |
| N-Acetyl-L-glutamic acid | 0.125 | 0.077 | 0.184 | -0.219* | 0.091 |
| N6-Acetyllysine | 0.121 | -0.012 | 0.184 | -0.253* | 0.09 |
| Alpha-Hydroxyisobutyric acid | 0.216* | 0.118 | 0.330** | -0.323** | 0.028 |
| Choline | -0.062 | -0.183 | -0.064 | -0.045 | 0.205 |
| p-Hydroxyphenylacetic acid | 0.044 | 0.112 | 0.216* | -0.202 | -0.062 |
| Pantothenic acid | 0.042 | 0.074 | 0.230* | -0.204 | 0.065 |
| Homocitrulline | 0.085 | 0.021 | 0.239* | -0.169 | 0.006 |
| Methylsuccinic acid | 0.116 | 0.122 | 0.125 | -0.214* | 0.054 |
| D-Glucuronic acid | 0.116 | 0.075 | 0.266* | -0.236* | 0.106 |

## * p < 0.05，** p < 0.01

**Table S9. Transcriptomic and Proteomic Changes of Key Molecules in Blood Stasis Syndrome After YQHX Intervention**

| ID | Name | FC | *p* value | log2 FC |
| --- | --- | --- | --- | --- |
| P00734 | F2 | 0.826578514 | 0.0021179 | -0.274776231 |
| P00451 | F8 | 0.793391203 | 0.00445166 | -0.333895693 |
| P00740 | F9 | 0.781054847 | 0.000807362 | -0.356504235 |
| P01024 | C3 | 0.847586659 | 0.014720275 | -0.238567215 |
| P02751 | FN1 | 2.406140447 | 0.001277117 | 1.2667208552046 |
| ENSG00000117322 | CR2 | 0.894 | 0.485826654 | -0.166777579 |
| ENSG00000177455 | CD19 | 0.899 | 0.527489514 | -0.153524217 |
| ENSG00000012124 | CD22 | 0.9 | 0.574347746 | -0.150644203 |
| ENSG00000105369 | CD79A | 0.956 | 0.800587342 | -0.06308886 |
| ENSG00000007312 | CD79B | 1.101 | 0.532726069 | 0.138965288620014 |
